# Inducible Calling Cards: Developing Mouse Reagents for Experimentally Controlled Transposon Insertion In Vivo

**DOI:** 10.1523/ENEURO.0411-25.2026

**Published:** 2026-06-24

**Authors:** Simona Sarafinovska, Arthi Venkatesan, Titilope M. Akinwe, Alexander Chamessian, Pamela Recio, Xuhua Chen, Maria Payne, Meaghan C. Creed, Robi D. Mitra, Joseph D. Dougherty

**Affiliations:** ^1^Departments of Genetics, Washington University School of Medicine, St. Louis, Missouri 63108; ^2^Psychiatry, Washington University School of Medicine, St. Louis, Missouri 63108; ^3^Department of Anesthesiology, Washington University Pain Center, St. Louis, Missouri 63108; ^4^Edison Family Center for Genome Sciences and Systems Biology, Washington University School of Medicine, St. Louis, Missouri 63108; ^5^Intellectual and Developmental Disabilities Research Center, Washington University School of Medicine, St. Louis, Missouri 63108

**Keywords:** genomic recording, immediate early genes, inducible system, mouse models, transposase

## Abstract

The piggyBac transposase enables robust forward genetic screens in mice. Furthermore, fusions of piggyBac with specific transcription factors (TFs) enables recovery of recoverable transposons (Calling Cards) for recording location of DNA binding events in vitro and in vivo. Such applications would be enhanced by engineering inducible transposases, such that timing of recording could be precisely controlled in vivo, as has been previously developed in vitro*.* Here, we tested two approaches for applying inducible Calling Cards (iCC) in the murine brain. We engineered knock-ins of inducible versions of two TFs: Jun, an immediate early gene that serves as a proxy for neural activity, and Sp1, a promiscuous binder of CpG unmethylated regions that indicates active promoters. We fused hyperPiggyBac transposase and a tamoxifen (TAM)-inducible domain (ERT2) to these TFs and tested the system for efficacy and temporal control of insertions, both in vitro and in vivo. Male and female Jun-iCC mice developed normally with no behavioral abnormalities showed TAM-dependent recording and captured neural activity during pharmacologically induced seizures. Jun-iCC yields relatively low numbers of insertions, likely due to the transient expression of Jun. In contrast, Sp1-iCC provided substantially higher insertion numbers, but transgenic animals of either sex exhibited developmental abnormalities, including reduced viability, anophthalmia, and reduced body weight, suggesting that ERT2 domains may sequester Sp1 and thus significantly impact development. Nonetheless, these iCC mouse lines enable drug-inducible integration of transposon cargo into specific loci in the living mouse.

## Significance Statement

Transposases, like the more widely used recombinases (Cre, Flp, etc.), enable the integration of a variety of DNA sequences into the genome, but without dependence on LoxP sites. However, few mouse reagents exist for transposases. Cre has been adapted to be drug-inducible, as well as activity-regulated (e.g., FOS-TRAP). Thus, additional applications would become available if transposases could also be drug-controlled and activity-dependent. Furthermore, there may be benefits to targeting integration to specific genomic region types (e.g., promotors). Here, we report results testing mouse lines for two drug-inducible transposase fusions to transcription factors (TFs)—the promoter-binding SP1 and activity-dependent Jun. Both allowed genomic integration, with viability, efficiency, inducibility, and targeting varying by TF fusion.

## Introduction

Transposons have wide applications for genomics and synthetic biology. PiggyBac, the cut-and-paste transposase derived from *Trichoplusia ni*, excises transposons with inverted terminal repeats and inserts them, together with up to 15 kb of DNA cargo, into DNA. It requires only long terminal repeats within the donor transposon and can insert into any available TTAA sequence (Cadiñanos and Bradley, 2007; Troyanovsky et al., 2016). PiggyBac has been further engineered to increase transposition activity, leading to hyperpiggyBac (hyPB; Cadiñanos and Bradley, 2007). PiggyBac and derivatives have been utilized in mammalian systems for the insertion of expression constructs to create cell lines and the insertion of gene traps for genetic screens (Rad et al., 2010; Chew et al., 2011; Troyanovsky et al., 2016). For instance, by crossing mouse lines with hyperpiggyBac engineered into the Rosa26 locus with mouse lines carrying arrays of transposons, novel tumor suppressors were identified in a large-scale forward genetic screen (Rad et al., 2010).

Notably, these transposases have affinity for the BRD4 chromatin modifier, which localizes to enhancers. Thus, we have used engineered transposons (“Calling Cards”), recovered by high-throughput sequencing, to map hyPB activity and, thus, BRD4 binding (Cammack et al., 2020; Moudgil et al., 2020; Yen et al., 2023, Guo et al., 2024). Furthermore, fusions of hyPB to transcription factors (TFs) can redirect transposase activity to other loci. This enables an alternative to Cut-N-Run or ChIP-Seq for assessing the binding pattern of specific TFs in the brain, with one additional, unique feature—the Calling Card is a permanent genomic mark of transient DNA binding events. Thus, Calling Cards have potential applications for associating historical epigenetic events with later cellular outcomes (Witchley et al., 2021; Boros et al., 2025).

**Figure 1.**
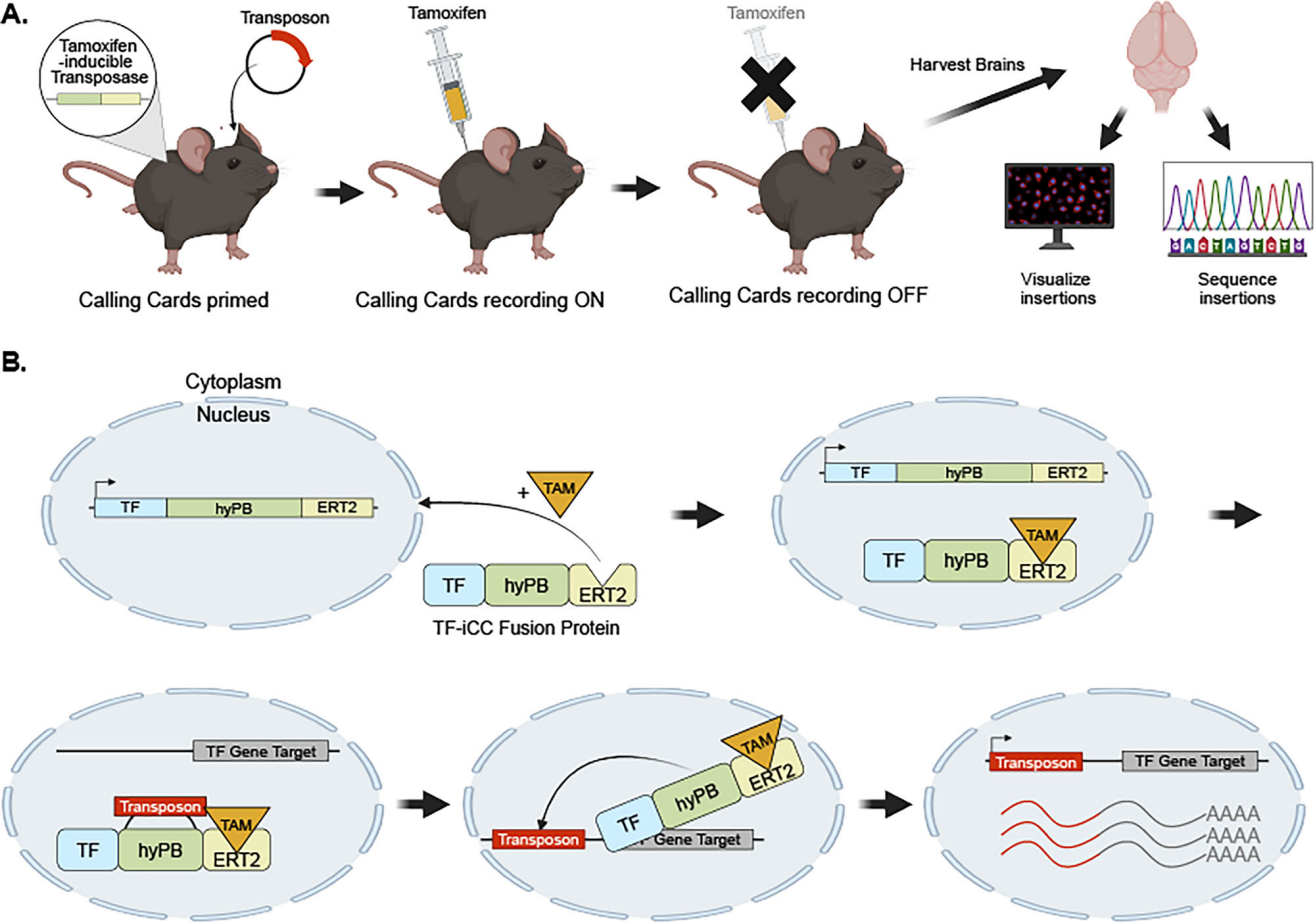
iCC would allow temporally controlled recording of transient molecular states. ***A***, The iCC system contains two components: a mouse line with a TAM-inducible transposase knocked into an endogenous locus and a virally delivered transposon. The presence of these two components “primes” Calling Cards for recording of transposon insertions to turn ON upon systemic delivery of TAM (or its metabolites) for the desired amount of time. Calling Cards recording “turns OFF” with clearance of TAM from the system. The recorded transposon insertions can be visualized using immunofluorescence or sequenced via next-generation sequencing of the brain tissue from the killed animal. ***B***, At the molecular level, the Calling Cards transposase, hyPB, is linked to a TAM-inducible domain, ERT2, which are knocked into a TF of interest. With transcription and translation of the TF, the TF-hyPB-ERT2 (TF-iCC) fusion protein is produced in the cytoplasm but unable to enter the nucleus due to the unbound conformation of ERT2. TAM binding allows nuclear translocation. In the nucleus, hyPB can bind to one of the virally delivered transposon sequences. Upon TF-iCC protein binding to a TF gene target, hyPB integrates the transposon into nearby DNA, creating a permanent record of the transient TF-DNA binding event. If SRTs are used, as the transposon has a strong promoter site, it harnesses transcription machinery to “self-report” its location in the genome via mRNA which can then be sequenced with a transposon-selective RNAseq protocol. Created in Biorender.

One drawback of existing CC tools, however, is that once delivered to the brain, they continuously record until the animal is killed; there is no ability to more precisely control the timing of CC activity. To overcome this limitation, we and others previously developed inducible Calling Cards (iCC), using standard drug-controllable systems [e.g., tamoxifen (TAM)] in culture (Qi et al., 2017). Here, we test two mouse lines for iCC in vivo. We utilize a TAM-inducible domain, ERT2 (Donocoff et al., 2020), to confer inducibility to Calling Cards recording, engineering hyPB-ERT2 fusions of two TF loci in the genome. Cytoplasmic heat shock protein 90 (HSP90) binds to the ERT2 domain, sequestering the fusion protein to the cytoplasm (Qi et al., 2017). The presence of TAM abolishes HSP90 binding, which in turn allows the fusion protein to enter the nucleus, and thus “turns on” Calling Cards recording (Fig. 1).

We have previously adapted the DNA binding domain of SP1 to AAV CC (Cammack et al., 2020). Sp1 is a ubiquitously active TF that binds to unmethylated CpG sites as a proxy for actively transcribed regions (Li et al., 2004). Here, we engineered a knock-in, which enables tagging the full-length protein and adding the ERT2 domain, circumventing existing size limitations of AAV. Sp1-iCC provides a readout of actively transcribed regions during the TAM delivery window, serving as a recorded proxy for gene expression.

We also sought to test whether CC could be adapted to be activity-dependent as well as drug-dependent, inspired by targeted recombination in active populations (TRAP; Guenthner et al., 2013). TRAP has allowed such recording for circuit-level analysis: using a TAM-inducible Cre recombinase whose expression is driven by an immediate early gene (IEG) promoter, such as *Fos* or *Arc*, TRAP allows recombination and expression of reporter proteins, only in the presence of both TAM and neural activation (Guenthner et al., 2013). Thus, we targeted Jun, an IEG and binding partner of Fos at Activator Protein 1 (AP-1) sites (Rauscher et al., 1988). Much like Fos-TRAP, the Jun-iCC construct should require both TAM and neural activity for recording to occur. Thus, Jun-iCC, should tag activated neurons with a fluorescent reporter, providing an orthogonal approach to TRAP. Uniquely, Jun-iCC could also provide a postmortem readout of AP-1 binding during the specified time window, thus serving as a proxy for neural activity-dependent gene expression.

We found that both constructs were TAM-inducible, and we were able to successfully tag activated neurons with Jun-iCC. However, the number of recoverable insertions in Jun-iCC was low compared with AAV Calling Cards. With Sp1-iCC, we were able to recover many more recorded binding events; however, we observed a significant reduction in viability in the Sp1-iCC knock-in animals. Overall, these two mouse lines demonstrate the potential for temporally controlled recording of transcriptional states in vivo and encourage further development of iCC in the mouse genome.

## Materials and Methods

### In vitro validation of iCC

To validate TAM-dependent transposase activity, we transfected Jun-hyPB-ERT2 knock-in N2a cell lines with BrokenHeart (BH) reporter plasmid (4 µg, PRM1294) using Neon electroporation (1,050 V, 30 ms, three pulses). Cells were treated with 1 µM 4-hydroxy TAM (4-OHT) or vehicle control, and 16.8 µM propofol was used to induce Jun expression 24 h post-transfection (Kidambi et al., 2009). tdTomato fluorescence was assessed to confirm transposase activity.

For insertion site analysis, we transfected Jun-iCC cells with self-reporting transposon (SRT-PURO), treated with 4-OHT, and selected with puromycin (3 µg/ml). Wild-type (WT) N2a cells cotransfected with SRT-PURO and unfused hyPB served as controls. Propofol (16.8 µM) was added before cell harvest to induce Jun expression. Genomic DNA was extracted, and insertion sites were identified by next-generation sequencing, as below. We recovered 262,121 unique insertion events from Jun-iCC cells compared with 473,304 from controls. Insertion site specificity was analyzed using Homer motif enrichment analysis (Heinz et al., 2010).

Similar validation experiments were performed with Sp1-hyPB-ERT2 knock-in N2a lines using the BH reporter system to confirm 4-OHT-dependent transposition without propofol stimulation, as Sp1 activation does not require additional stimulus. In brief, two Sp1-iCC cell lines were transfected with BH as above and treated with 1 µM 4-OHT or vehicle control. tdTomato fluorescence was again assessed to confirm transposase activity. All cell culture experimental series were repeated a minimum of two independent times, with multiple clones as available, showing qualitatively similar results. Only the final experimental series was quantified for supplemental figures.

Using the ImageJ software, TIF images underwent contrast and brightness enhancement [setMinAndMax(0, 100)], Gaussian blurring (sigma, 5 scaled), and background subtraction (rolling ball radius, 200 pixels). thresBinary masks for bright-field and RFP channels were created using the “Make Binary” function, followed by pixel value normalization based on threshold settings and watershedding. The “Image Calculator” function was used to subtract these masks from the original fluorescence images, identifying RFP+ regions of interest (ROIs) within the cells. Cell counts were obtained using the “Analyze Particles” function. Once all images were identically processed, the statistical significance of differences by genotype was assessed using an ANOVA to test the effect of genotype.

### Generation of iCC mouse lines

CRISPR-Cas9-mediated homology-directed repair was used to generate TF-hyPB-myc-ERT2 knock-in mice. For Jun and Sp1 targeting, guide RNAs were designed and evaluated for off-target effects, with optimal candidates selected based on proximity to target sites (5′ CGTTTTGAGAACAGACTGTCNGG 3′). Donor templates contained left and right homology arms flanking the hyPBase-myc-ERT2 cassette, with Sp1 constructs including an 18aa linker sequence (5′ CAAGTGGGCGGCGGCGCGCCCCGCCTGGGCGGCGGACCCAAGC 3′). Junction PCR primers verified correct integration: for Jun, 5′ junction (F, CTCATACCAGTTCGCACAGGC; R, GGATGTGCTCGTCGTCCAGG) and 3′ junction (F, CTGCGGGCTCTACTTCATCG; R, GGCCCATCTCTCTTGTGACTCTG) yielded 1,612 and 1,675 bp products, respectively; for Sp1, 5′ junction (F, CTCATACCAGTTCGCACAGGC; R, GGATGTGCTCGTCGTCCAGG) and 3′ junction (F, CTGCGGGCTCTACTTCATCG; R, GGCCCATCTCTCTTGTGACTCTG) yielded 1,360 and 1,656 bp products, respectively. Following validation in N2A cells, fertilized oocytes were electroporated with verified gRNAs, Cas9 protein, and donor templates. Random integration was assessed with TF-specific primers for both 5′ and 3′ integration sites: for Jun, 5′ (F, AACCTTGAAAGCGCAAAACTCCGA; R, GGATGTGCTCGTCGTCCAGG) and 3′ (F, CTGCGGGCTCTACTTCATCG; R, CACTGGGGGCGCCGTCAGGTC); for Sp1, 5′ (F, CAGAACAAGAAGGGAGGCCC; R, GGATGTGCTCGTCGTCCAGG) and 3′ (F, CTGCGGGCTCTACTTCATCG; R, AGTGACATTGGGTGCCACAA). PCR conditions employed SuperFi polymerase with GC enhancer, with specific cycling parameters optimized for each junction. For Jun targets, cycling conditions were 98°C for 2 min, 37 cycles of 98°C for 20 s, 64°C for 30 s, and 72°C for 2 min, followed by 72°C for 5 min. For Sp1 targets, 5′ junction PCR used SuperFi at 65°C with GC enhancer, while 3′ junction PCR used SuperFi at 64°C with a 69°C/3:00 extension. Successful founders with targeted integration at both junctions were identified by PCR and Sanger sequencing, including Jun-targeted males M2, M8, M34, and M45 and females F1, F28, F30, and Sp1-targeted males M5 and M9. Each line was validated for viability and fertility to choose the final candidate line. For both lines, the sperm from two proven male breeders were cryopreserved and stored in two separate locations for security.

### Animals

All animal studies were approved by and performed in accordance with the guidelines of the Animal Care and Use Committee of Washington University in Saint Louis, School of Medicine, and conform to NIH guidelines of the care and use of laboratory animals. The animals were housed in controlled environments with a 12 h light/dark cycle, constant temperature and relative humidity, and *ad libitum* access to food and water. Heterozygous males for Sp1-iCC and Jun-iCC were crossed to our in-house C57BL/6J females for breeding. The transgenic lines were refreshed every 8–10 generations by backcrossing to freshly obtained C57BL/6J males and females from Jackson Laboratory (Strain Number 000664). Upon weaning at Postnatal Day (P)21, the animals were group-housed by sex, genotype, and treatment, where applicable. Mouse lines are donated to the JAX MMRRC for distribution via stock numbers 76120 and 76121 for Jun-ICC and Sp1-ICC, respectively.

### Genotyping

The genotyping tissue (tail biopsy, ear punch, or toe clipping) was obtained from each animal and placed in a PCR tube. A 100 µl lysis buffer (25 mM NaOH, 0.2 mM EDTA), pH 12, was added to each tube and incubated at 99°C for 60 min in a thermocycler. Once the samples cooled to room temperature, 100 µl 40 mM Tris–HCl, pH 5, was added to neutralize the alkaline lysis buffer. The crude lysate containing genomic DNA (gDNA) was stored at 4°C. All genotyping PCRs were multiplexed with β-actin_For (AGAGGGAAATCGTGCGTGAC) and β-actin_Rev (CAATAGTGATGACCTGGCCGT) primers, as this not only confirms the presence of gDNA but also minimizes nonspecific amplification. Common hyPB primers were used for both Jun-iCC and Sp1-iCC genotyping (F, TGATGACCTGCAGCAGAAAG; R, GCTGATGTTGTCCCTCAGGT). For each reaction, 1 µl crude gDNA was mixed with 5 µl OneTaq Quick-Load 2× Master Mix (New England Biolabs M0271), 1 µl 10 µM hyPB For/Rev primer mix, 0.25 µl 10 µM β-actin For/Rev primer mix, 2 µl Betaine (Thermo Fisher Scientific J77507.UCR), and 0.75 µl ddH2O. PCR products were run on a 1% agarose gel and visualized with GelRed (Biotium 41003).

### Protein expression analysis of Jun-iCC fusion protein

To visualize nuclear translocation of Jun-hyPB-ERT2 (iCC), we used Jun-iCC and Jun-WT littermates. Animals (*n* = 3 per group) received a single dose of TAM (100 mg/kg) or vehicle control via intraperitoneal injection. Dorsal root ganglia (DRGs) were harvested 12 h postinjection. Proteins were extracted using RIPA buffer supplemented with protease inhibitors, quantified by BCA assay, and resolved on 4–15% gradient SDS–PAGE gels. Western blotting (i.e., simple Western capillary electrophoretic immunoassay) was performed using antibodies against c-Jun (rabbit anti-c-Jun 60A8, Cell Signaling Technology 9165, at 1:50) to detect both WT Jun (∼40 kDa) and the Jun-iCC fusion protein (∼180 kDa, calculated molecular weight 144 kDa). The presence of bands at expected molecular weights confirmed expression of both proteins in the appropriate genotypes, with the Jun-iCC fusion protein detected exclusively in the iCC samples. Smearing reflected disproportionally high signal between bands; thus, reduced contrast enhancement was performed to produce clearer single bands.

### Open field

Behavioral assays to evaluate effects of Jun-iCC knock-in on animal behavior were conducted in *N* = 44 animals, split into two batches (Batch 1 *n* = 20; Batch 2 *n* = 24). Both batches were split by genotype (*N*_Jun-iCC_ = *22*; *N*_Jun-WT_ *=* 22) and sex (*N*_female_ = 24; *N*_male_ = 20). All tasks were run during the light phase, by a female experimenter blinded to the groups tested.

The 1 h open-field (OF) test was used to assess the general activity, exploratory behavior, and emotionality of the mice (*N*_total_ = 44; *N*_batch 1_ *=* 20; *N*_batch 2_ *=* 24; *N*_female_ = 24; *N*_male_ = *20*; *N*_Jun-iCC_ = 22; *N*_Jun-WT_ *=* 22). The task was administered at P47 for Batch 1 (range P44–51) and P62 for Batch 2 (range P60–64). We performed the protocol similarly to our published work (Chaturvedi et al., 2024). The apparatus consisted of matte white acrylic enclosures (40 × 40 × 30 cm high) enclosed within a white sound-attenuating chamber (70.5 × 50.5 × 60 cm), with red 9 lux illumination (LED Color-Changing Flex Ribbon Lights, Commercial Electric). Animals were habituated to the testing room for 30–60 min before testing. Each animal was placed in the center of the maze and allowed to explore freely for 1 h. Behavior was recorded using ANY-maze (Stoelting), which established a 28.3 × 28.3 cm central zone (50% of total area) and a bordering 5.9 cm peripheral zone, which are delineated on the computer program only. Outcomes included the number of entries into and time spent in the center as well as the distance traveled in each zone. The apparatus was cleaned with 0.02% chlorhexidine solution (Nolvasan, Zoetis) between animals. One animal from Batch 1 and three animals from Batch 2 were excluded from OF analyses due to jumping.

### Sensorimotor battery

Balance, strength, coordination, and motoric initiation were assessed by a battery of sensorimotor measures (SMB) using the protocols described in Minakova et al. (2021). The series of tasks was administered at P49 for Batch 1 (range P46–53) and P69 for Batch 2 (range P67–71). The battery included walking initiation, ledge, platform, pole, and inclined and inverted screen tests. For each test, the experimenter manually recorded with a stopwatch. Two trials were conducted for each test, back-to-back, and the average was used for the analyses. Tests are divided into 2 d and are not counterbalanced to provide each animal with the same testing conditions. The first day of testing consisted of the walking initiation, ledge, platform, and pole tests. The second day of testing consisted of the inclined and inverted screen tests. Each test was conducted for a maximum of 60 s, except for the pole test, which lasted for a maximum of 120 s. Animals were habituated to the testing room for 30–60 min before testing.

Walking initiation was evaluated by placing the mouse on a flat surface inside a square measuring 24 × 24 cm and recording the time it takes the mouse to leave the square (i.e., all four limbs have crossed the square at the same time). The ledge and platform tests were used to assess basic balance ability. The ledge test requires the mouse to be placed on a Plexiglas ledge measuring 0.5 cm deep and standing 38 cm high. The time the mouse is able to balance on the ledge is recorded. During the platform test, the mouse is placed on a wooden platform measuring 3.5 cm thick and 3.0 cm in diameter and elevated 25.5 cm above the base. The time the mouse was able to balance on the platform was recorded. Fine motor coordination was elevated by the pole test, where the mouse is placed head upward on a vertical pole with a finely textured surface. The time the mouse takes to turn downward 180° and climb to the bottom of the pole was recorded. The 60°, 90°, and inverted screen tests are used as measures of strength and coordination. During the inclined screen tests, the mouse is placed head oriented downward in the middle of a mesh wire grid measuring 16 squares per 10 cm, elevated 47 cm, and inclined to 60 or 90°. The time the mouse spends turning upward 180° and climbing to the top of the screen is recorded. For the inverted screen test, the mouse is placed head oriented downward in the middle of a mesh wire grid measuring 16 squares per 10 cm, elevated 47 cm, and when it is determined that the mouse has a proper grip on the screen, it is inverted to 180°. The time the mouse is able to hold on to the screen without falling off is recorded. The apparatuses were cleaned with a 70% ethanol spray between animals and between trials if excrement was in the way.

### Elevated plus maze

The elevated plus maze (EPM) was used to assess anxiety-like behavior by measuring avoidance of open spaces. The task was administered at P83 for Batch 1 (range P80–87) and P94 for Batch 2 (range P92–96). We performed the protocol as described previously (Nygaard et al., 2023). The apparatus (Kinder Scientific) consisted of two open arms and two closed arms arranged in a plus configuration and elevated above the floor. Animals were habituated to an adjacent room for 30–60 min prior to testing. Testing was conducted in darkness with each animal placed in the center of the maze and allowed to explore freely for 5 min. Behavior was recorded using the Ethovision software (Noldus). Measures included the number of entries into and time spent in open arms, closed arms, and center zone, as well as distance traveled in each zone. The apparatus was cleaned with 0.02% chlorhexidine (Nolvasan, Zoetis) solution between animals.

### Odorant holeboard

The holeboard (HB) exploration/olfactory preference task was used to evaluate olfaction, social preferences, and exploratory behavior. The task was administered at P51 for Batch 1 (range P48–55) and P73 for Batch 2 (range P71–75). We used the protocol adapted from Maloney et al. (2019). The apparatus is a computerized HB (41 × 41 × 38.5 cm) with eight equidistant holes in the floor (Learning Holeboard; MotorMonitor, Kinder Scientific). Beam breaks quantified the frequency and duration of holepokes (2 cm deep) and dips (1 cm deep). Animals were habituated to the testing room for 30–60 min before testing. Each animal completes the test over 2 d. On the first day, each mouse was given a 30 min habituation session during which the holes contained no odorants. The following day, a 20 min test session was conducted with three corner holes baited, with (1) a familiar odorant (corncob bedding soiled with urine and scent markings from same-sex mice), (2) a novel odorant (corncob bedding soiled with urine and scent markings from opposite-sex mice), and (3) a novel, putatively rewarding odorant (vanilla extract diluted onto a filter). The odorants were contained in a cup at the bottom of the hole (7 cm deep), and access was blocked by a plastic mesh cap. The configuration of the odorant-containing and empty corner holes was counterbalanced within and across groups. Exploration was quantified by measuring total ambulation. Olfactory preference was assessed by comparing holepoke frequencies between odorant-containing and empty corner holes. Specifically, we focused on comparing social holepoke frequencies between the same-sex bedding odorant and the opposite-sex bedding odorant. All odorant-containing cups were cleaned with mild soap and water, and the entire apparatus was cleaned with a 0.02% chlorhexidine (Nolvasan, Zoetis) solution. During Batch 1 testing, a malfunction in one of the three apparatuses prevented the recording of habituation and test session data for seven mice.

### Statistical analysis

Behavioral statistical analyses were performed using the IBM SPSS Statistics software (v.28). Prior to analysis, data were screened for missing values and checked for ANOVA assumptions within levels of genotype, sex, batch, and their interactions. Normality was assessed using Shapiro–Wilk tests on *z*-score–transformed data, along with histograms and *Q*–*Q* plots. Levene's test evaluated the homogeneity of variances. Outliers were identified via boxplots and *z*-scores exceeding ±3.00. When assumptions were violated, nonparametric Mann–Whitney *U* tests replaced ANOVAs. The probability value for all analyses was *p* < 0.05.

### Intracerebroventricular injections

Injections were performed as described in the intracerebroventricular injection section within Basic Protocol 1 found in Yen et al. (2023). Briefly, the pups were anesthetized on ice, and a total of 6 µl (3 µl per hemisphere, 1 µl per site) was injected into the ventricles of P0–1 pups using a 50 µl Hamilton syringe. After the injections, the pups were kept warm on a heating pad until they were returned to their home cage.

### TAM administration

For temporal control of the iCC system, TAM (Sigma-Aldrich T5648) was administered via intraperitoneal injection. The TAM working solution (10 mg/ml) was prepared by dissolving 50 mg TAM in 5 ml of vehicle solution consisting of sunflower oil (Sigma-Aldrich) and 100% ethanol at a 9:1 ratio. The mixture was vortexed for 30–60 min with parafilm-sealed tubes until completely dissolved. Vehicle control solution (Veh) consisted of the sunflower oil and ethanol mixture without TAM. Fresh working solutions were prepared weekly and stored at 4°C.

For induction experiments, mice were weighed prior to the first dose and administered 100 mg/kg TAM (or equivalent volume of vehicle) daily for 5 consecutive days, alternating sides of the intraperitoneal cavity to minimize irritation. All injections were performed using 23 gauge needles to accommodate the viscous solution. TAM-treated and Veh-treated animals were housed separately to prevent cross-contamination through coprophagia. For all experiments, animals were killed at least 7 d after the final TAM dose to allow for clearance of the drug and completion of the recording window.

### Immunofluorescence and imaging

Animals were deeply anesthetized with isoflurane in an induction chamber until unresponsive to toe pinch. The right hemisphere was processed as per *Bulk Calling Cards library* preparations (Yen et al., 2023). The left hemisphere was harvested and drop-fixed in a tube containing 4% (w/v) PFA overnight at 4°C. Then the tissue was cryoprotected in 15% (w/v) sucrose, then 30% (w/v) sucrose at 4°C, and then frozen in plastic molds (Polysciences 18646A-1) containing OCT compound (Thermo Fisher Scientific 23-730-571). The tissue blocks were kept at −80°C until further processing. The tissue was cut into 35-µm-thick sagittal or coronal free-floating sections for immunostaining. The sections were permeabilized with 0.1% (v/v) Triton X-100 for 15 min and blocked with 5% (v/v) normal donkey serum (Jackson ImmunoResearch Laboratories 014-000-121) for 60 min. The primary antibodies used were rabbit anti-RFP (Rockland Immunochemicals 600-401-379) at 1:500 dilution, rabbit anti-c-Jun (60A8; Cell Signaling Technology 9165), and mouse anti-TUBB3 (BioLegend 801213) at 1:1,000 dilution. Secondary antibodies used were donkey anti-rabbit Alexa Fluor 568 (Thermo Fisher Scientific A10042), donkey anti-rabbit Alexa Fluor 488 (Thermo Fisher Scientific R37118), and donkey anti-mouse Alexa Fluor 488 (Thermo Fisher Scientific A21202) at 1:500 dilution. A 1 µg/ml DAPI (Thermo Fisher Scientific D1306) was used to stain the nuclei blue. Sections were mounted onto slides with ProLong Gold Antifade mounting medium (Thermo Fisher Scientific P36934) and sealed with nail polish. High-magnification confocal images were captured using 20× or 40× objectives on the LSM700 AxioImager Z2 (Zeiss). For quantifying the RFP+ area in pixels (Extended Data Figs. 5-1, 6-1), at least two mice per condition and one section per mouse were analyzed.

Using the ImageJ software, TIF images underwent contrast and brightness enhancement [setMinAndMax(0, 100)], Gaussian blurring (sigma, 2 scaled), and background subtraction (rolling ball radius, 20 pixels). thresBinary masks for the RFP channel were created using the “Make Binary” function, followed by pixel value normalization based on threshold settings and watershedding. The “Image Calculator” function was used to subtract this mask with the original fluorescence images, identifying RFP+ ROIs. Cell counts were obtained using the “Analyze Particles” function. Once all images were identically processed, the statistical significance of differences by genotype was assessed using an ANOVA to test the effect of genotype.

### Bulk Calling Cards library preparations

Tissue homogenization, RNA isolation, and library preparation steps are described in Basic Protocol 2 and 3 found in Yen et al. (2023). Briefly, the dissected brain tissue was cut into five chunks to identify up to five independent insertion events at any given insertion locus, snap-frozen in the vapor phase of liquid nitrogen, and then stored at −80°C until further processing. For homogenization, the tissue chunk homogenized in Trizol Reagent (Thermo Fisher Scientific 15596018) and total RNA was harvested using the RNA Clean & Concentrator Kit-25 (Zymo Research R1018) with slight modifications as described in Yen et al. (2023). Bulk sequencing libraries were generated and sequenced on the Illumina platform. Calling Cards found at the same insertion site were considered distinct if they had distinct barcodes (i.e., came from different tissue chunks). Insertions that pass filtering were treated equally during analysis, regardless of read depth.

### Sequencing

Pooled dual indexed libraries were submitted to the Genome Technology Access Center at the McDonnell Genome Institute (GTAC@MGI) for sequencing. For their workflow, the concentration of each library was determined using the KAPA Library Quantification Kit according to the manufacturer's protocol. Target sequencing depth was determined prior to pooling, and samples were pooled in ratios based on the targeted depth and concentrations to produce cluster counts appropriate for the Illumina NovaSeq 6000 instrument. Normalized libraries were sequenced on a NovaSeq 6000 S4 Flow Cell using the XP workflow and a 151 × 10 × 10 × 151 sequencing recipe according to the manufacturer's protocol. Base calls were converted to fastq format and demultiplexed using the onboard DRAGEN software to run BCL Convert.

### Bulk Calling Cards analysis

The raw FASTQ files were processed using the pipeline found at https://github.com/Dougherty-Lab/inducible_calling_cards. The resulting qbed files were filtered to keep only insertions with >2 reads. Motif enrichment analysis was used from the HOMER suite of tools (Heinz et al., 2010). Common genome arithmetic operations such as merging, intersecting, and counting genome regions were performed using the Bedtools utilities (Quinlan and Hall, 2010).

### Pentylenetetrazol administration and seizure monitoring

To induce neural activity for in vivo testing of the iCC system, we administered pentylenetetrazol (PTZ; a GABA antagonist) to mice (Van Erum et al., 2019). PTZ solution (5 mg/ml) was prepared fresh on the day of use by dissolving 50 mg PTZ in 10 ml of sterile 1× PBS. The mixture was vortexed and incubated in a 37°C water bath for ∼10 min to ensure complete dissolution.

Mice were weighed immediately before injection to calculate the appropriate dose volume. For all experiments, we used a 30 mg/kg dose, calculated as follows: (0.030 × body weight in grams × 1,000)/5 = injection volume in microliter. PTZ was administered via intraperitoneal injection, and animals were immediately placed in a clean observation cage with fresh bedding for seizure monitoring.

Seizure activity was scored according to the modified Racine scale (Racine, 1972; Van Erum et al., 2019). For our experiments, we recorded the latency to freezing and monitored animals for a minimum of 15 min. All animals displayed mild seizures that did not progress to more severe stages. Following seizure induction, animals were returned to their home cages after regaining normal locomotion and monitored daily for any adverse effects.

### Data and code availability

Raw data and processed data are available upon request. Code used to analyze the data and complete documentation can be found on the Dougherty-Lab/inducible_calling_cards main page (https://github.com/Dougherty-Lab/inducible_calling_cards).

## Results

### Jun-iCC is inducible in vitro

First, we engineered a Jun-iCC knock-in N2a cell line for in vitro validation. We chose Jun as our initial TF target to complement existing FosTRAP technologies that utilize IEGs to capture neural activity across development or in response to behavioral stimuli (Cammack et al., 2020). Into the endogenous Jun locus, we engineered the hyperPiggyBac (hyPB) transposase, a myc tag, and an ERT2 domain (Yusa et al., 2011). The ERT2 domain is a humanized estrogen receptor that sequesters any fusion protein to the cytoplasm, allowing nuclear translocation only in the presence of TAM or TAM metabolites (Indra et al., 1999; Donocoff et al., 2020). Thus, the Jun-hyPB-myc-ERT2 fusion protein should only be transcribed following neural activity and in the presence of TAM metabolites, conferring temporal control to activity-dependent hyPB transposase insertions. Junction PCR and Sanger sequencing of Jun-hyPB-myc-ERT2 region confirmed that the insertion was successful, and there were no mutations in any of the domains.

To assess whether Jun-iCC recording in the N2a cell line is TAM metabolite-dependent, we designed a series of validation experiments. First, we transfected the BrokenHeart (BH) transposon into Jun-iCC cells, which provides a low-background fluorescent readout of transposase activity (Extended Data Fig. 5-1*A*; Cammack et al., 2020). Our results demonstrated that hyPB insertions almost exclusively occur only in the presence of 4-hydroxytamoxifen (4-OHT), a TAM metabolite (Extended Data Fig. 5-1*B–D*), confirming the inducibility of our system. We observed that neural activity, induced by propofol, did not visibly affect insertion rates in the Jun-iCC line, likely because IEGs like Jun are more ubiquitously expressed in tumor cell lines than at baseline in the mouse brain (Angel et al., 1988). We note that, as expected, knock-in Jun-fusions have much lower activity than native TdTomato expressed from a strong constitutive promoter knocked into the HEK 293T genome or transfected on a plasmid in the N2A-Jun cell line (PRM1051).

We next sought to assess the quantity and positions of the insertions and confirm the system would work with the tools we had already developed for in vivo CC. We have previously adapted the Calling Card system to function in vivo, by delivering both the transposase and transposons using adeno-associated virus (AAV) vectors and have shown its ability to record across mouse brain development (Cammack et al., 2020; Moudgil et al., 2020; Yen et al., 2023). In the mouse brain, CC relies on two components: a hyPB transposase, which can be delivered virally or knocked into the genome, and an engineered self-reporting transposon (SRT), which is delivered virally. Therefore, to prepare for in vivo testing of these new mouse lines constructs, we also sought to benchmark our cell knock-ins with SRTs. The SRT contains an elongation factor-1 alpha (EF1α) promoter, which allows it to engage transcription machinery to transcribe mRNA, which will include the SRT sequence and portion of the flanking genomics sequence, until a cryptic polyadenylation site is reached (Moudgil et al., 2020). Thus, the SRT “self-reports” its location in RNA: via next-generation sequencing and alignment of the gene target on the mRNA to known gene loci, the location of TF(-hyPB)-DNA binding events can be determined. SRTs vastly increase the sensitivity for insertion sites compared with DNA-based recovery methods. The SRT additionally contains a tdTomato red fluorescent protein, which allows for immunofluorescent visualization of cells where active CC recording events occurred. Therefore, we next transfected the SRT into the cell lines to be able to read out Jun-iCC insertions via next-generation sequencing (Moudgil et al., 2020). Via bulk next-generation sequencing and Homer motif enrichment analysis, we show an expected enrichment for the Jun motif from in vitro Jun-iCC recordings, suggesting that the hyPB was indeed increasingly targeted to AP-1 binding sites (Extended Data Fig. 5-1*E*). Notably, the quantity of insertions, was lower in all Jun-iCC lines compared with the unfused hyPB positive control, with a twofold difference in insertion number by sequencing (262,121 in Jun-iCC lines vs 473,304 insertions in the positive controls), suggesting that the endogenous knock-in approach results in reduced Calling Cards recording efficiency, perhaps because of the fusion of the proteins, or because Jun expression may be more difficult to capture due to its transient nature.

### Jun-iCC mice are viable and does not disrupt behavior in vivo

**Figure 2.**
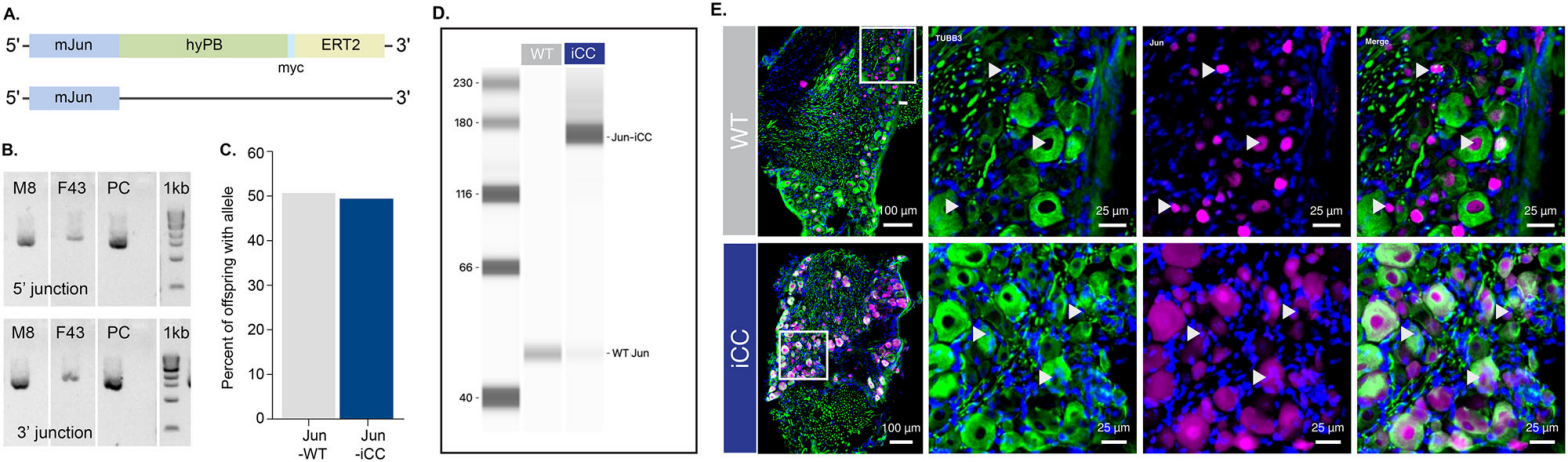
iCC knock-in to Jun is viable. ***A***, Schematic of the Jun alleles of the Jun-iCC mouse line. Jun-iCC had one normal copy of Jun, and one copy fused to the Calling Card transposase, hyPB, a myc tag, and the TAM-inducible ERT2 domain. ***B***, Junction PCRs showing that both M8 and F43 knock-in lines have expected integration at the Jun locus. ***C***, Bar chart showing the percentage of offspring with either Jun-WT or Jun-iCC allele. Expected inheritance (∼50%) of Jun-iCC allele in *N* = 179 offspring suggests that Jun-iCC does not impact viability. ***D***, DRG samples from Jun-WT mice (with two normal Jun alleles) and Jun-iCC mice (with hyPB-myc-ERT2 knock-in at one Jun allele) were analyzed by Western blot. The WT Jun protein (∼40 kDa) is detected in both genotypes, while the larger Jun-iCC fusion protein (∼180 kDa) is observed exclusively in the iCC samples. Molecular weight markers (kDa) are indicated on the left. This confirms expression of the Jun-hyPB-ERT2 fusion protein in the Jun-iCC mouse model. ***E***, Immunofluorescence of Jun in DRG sections from Jun-WT (top) and Jun-iCC mice (bottom). Anti-Jun antibody (magenta), anti-TUBB3 (green), and DAPI nuclear stain (blue). The right panels show enlarged boxed regions. Arrows indicate the location of nuclei labeling observed with DAPI. In Jun-WT, Jun localizes primarily to nuclei, while iCC samples show both nuclear and cytoplasmic staining (colocalization with TUBB3), confirming ERT2-mediated cytoplasmic retention without TAM. TUBB3, beta-tubulin 3. Scale bar, 25 µM; 100 µM.

Given promising in vitro data, we targeted the same Jun-iCC construct into mouse embryos to generate Jun-iCC founder mice: within the endogenous Jun locus, we engineered a hyPB-myc-ERT2 fusion protein, generating mice that are heterozygous for this mutation (Fig. 2*A*). We performed junction PCRs to confirm insertion sites and Sanger sequencing to verify that no unexpected mutations were introduced (Fig. 2*B*). We generated two founder lines, F43 and M8. We successfully bred these lines to the fourth generation with almost 200 offspring overall. Offspring showed expected Mendelian ratios (50% for heterozygous breeding), indicating that the Jun-iCC knock-in did not impact viability (Fig. 2*C*). Jun-WT indicates two normal copies of the Jun allele, while Jun-iCC indicates a heterozygous knock-in of Jun-hyPB-myc-ERT2 on one WT Jun allele.

Focusing on the M8 transgenic line for the remainder of the work, we confirmed the presence of the full Jun-hyPB-myc-ERT2 fusion protein. Since Jun is only transiently expressed in the brain, we evaluated this in the DRGs, where Jun expression is more durable (Buschmann et al., 1998). We found the WT Jun protein (∼40 kDa) in both Jun-iCC and Jun-WT genotypes, while the larger Jun-iCC fusion protein (∼180 kDa) was observed exclusively in the Jun-iCC samples (Fig. 2*D*). Immunofluorescence also showed a distinct localization pattern for the Jun-WT versus the Jun-iCC fusion protein, with Jun staining in the Jun-iCC mice displaying both normal nuclear and cytoplasmic localizations (Fig. 2*E*). This distinct cytoplasmic localization pattern in Jun-iCC mice confirms that the ERT2 domain is functioning as designed, sequestering the fusion protein in the cytoplasm until TAM administration triggers its translocation to the nucleus, a critical feature for achieving temporal control of the Calling Cards system.

**Figure 3.**
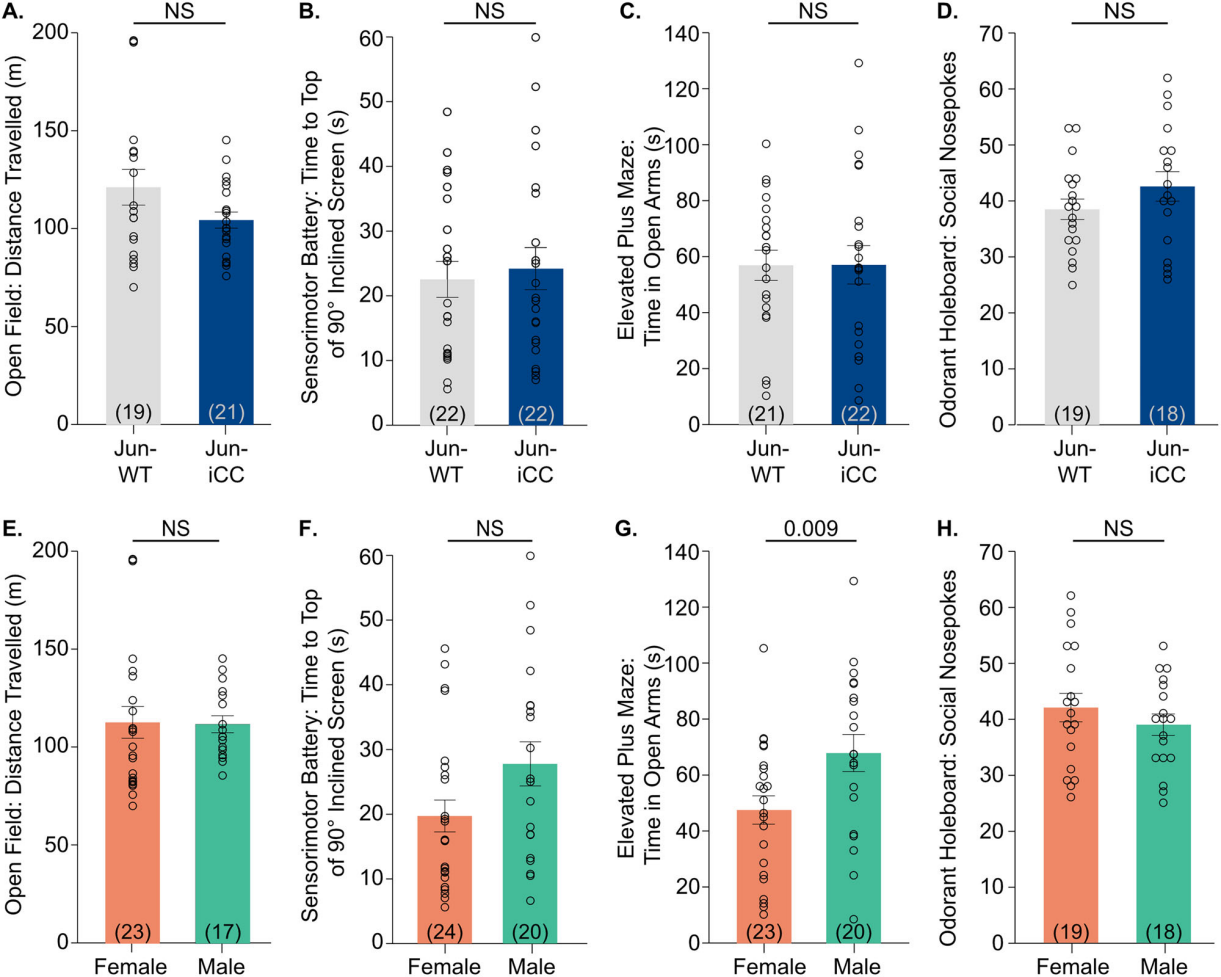
Jun-iCC mice show comparable behavior to Jun-WT littermates. ***A***, No significant differences were observed between Jun-iCC and Jun-WT mice in activity levels in OF. ***B***, Motor and balance ability in the sensorimotor battery was also intact in Jun-iCC mice. ***C***, Jun-iCC mice did not show increased avoidance in EPM. ***D***, iCC mice showed normal olfactory function and similar preference for social stimuli compared with Jun-WT littermates in the odorant hole-poking task. ***E***, No significant differences were observed between male and female mice (including both genotypes) in activity levels in OF. ***F***, Motor and balance ability in the sensorimotor battery was not significantly different between male and female mice. ***G***, Male mice spent more time in the open arms of the EPM than the female littermates (*p* = 0.009). ***H***, Male and female mice showed similar preference for social stimuli in the odorant hole-poking task. Error bars indicate mean ± SEM; individual dots show data from each animal. NS, not significant. No significant sex by genotype interactions were observed.

Finally, we tested whether the Jun-iCC mice displayed normal behavior (Table 1). In a cohort of 44 mice, evenly split by genotype, we tested locomotion, sensorimotor skills, anxiety, and olfaction. First, in the OF task, we observed no significant differences in activity levels or exploratory behavior between the two genotypes (Fig. 3*A*; Seibenhener and Wooten, 2015). There was no significant effect of sex on the ambulatory behavior of the animals (Fig. 3*E*), but we did find that males entered the center more frequently and spent longer there than females (Table 1). Next, in the sensorimotor battery, we found that motor and balance ability were intact in Jun-iCC mice relative to Jun-WT offspring (Fig. 3*B*). Jun-iCC animals did exhibit a slight reduction in platform, pole, and 60°-inclined screen tests compared with Jun-WTs (Table 1). On the platform test, Jun-iCC animals had a lower average time balancing on the platform than Jun-WTs animals, only in Batch 1. On the pole test, Jun-iCC animals had a lower average time on the pole than Jun-WT animals, only in Batch 2. On the 60°-inclined screen test, Jun-iCC animals had a higher average time to climb to the top of the screen than Jun-WTs animals, only in Batch 2. Notably, Jun-iCC animals performed normally on the 90° inclined screen and inverted screen tests, which are the most arduous tests of balance and strength, suggesting an overall normal phenotype in Jun-iCC mice relative to Jun-WT controls. In terms of sex effects overall, we saw that males had a lower average time balancing on the platform than females, only in Batch 1; other than that we found no effects of sex, notably with no difference between male and females in performance on the most arduous 90° inclined screen test (Fig. 3*F*). In the EPM, we found no differences in time spent in the open arms between genotypes, indicating that Jun-iCC mice do not show increased avoidance (Fig. 3*C*; Walf and Frye, 2007). Jun-iCC animals traveled less distance than Jun-WTs in the EPM, but they displayed normal exploratory behavior and activity in the OF, which is an overall better task to evaluate activity, given its hour-long testing duration and wider OF. For both open- and closed-arm entries, there was a significant group × batch interaction, driven primarily by batch effects, and Jun-WT animals entered the open and closed arms more frequently than Jun-iCC animals only in Batch 1. We also found a significant effect of sex, such that males entered the open arms more frequently and spent longer there than females (Fig. 3*G*; Table 1). Finally, in the odorant hole-poking task, we saw that Jun-iCC and Jun-WT mice both were able to distinguish different odors and had comparable numbers of nosepokes for a social odor, suggesting intact olfaction and similar preference for social stimuli (Fig. 3*D*; Wozniak et al., 2013). We also did not observe a significant effect of sex on the number of social nosepokes (Fig. 3*H*). In summary, we confirmed that a heterozygous iCC construct insertion at the endogenous Jun locus does not robustly impact fundamental behaviors, including locomotion, balance, avoidance, and olfaction.

**Table 1.** Statistical analysis of outcomes from Jun-iCC behavioral testing

Behavioral assay	Outcome	Model	Predictor	Output	*p* value
OF	Distance	Mann–Whitney	Group	*U*(*N*_Jun-WT_ = 19; *N*_Jun-iCC_ = 21) = 158; *z* = −1.124	*p* *=* 0.261
	Center entries	ANOVA	Sex	*F*_(1,32)_ = 12.305	*p* *=* 0.001
	Center time	ANOVA	Sex	*F*_(1,32)_ = 8.248	*p* *=* 0.007
SMB	Time on the platform	Mann–Whitney	Batch 1: Group	*U*(*N*_Jun-WT_ = 10; *N*_Jun-iCC_ = 10) = 30; *z* = −2.163	*p* *=* 0.031
			Batch 1: Sex	*U*(*N*_Jun-WT_ = 13; *N*_Jun-iCC_ = 7) = 28; *z* = −1.984	*p* *=* 0.047
	Time on the pole	Mann–Whitney	Batch 2: Sex	*U*(*N*_females_ = 11; *N*_males_ = 13) = 25; *z* = −2.694	*p* *=* 0.007
	Time to the top of 60° screen	Mann–Whitney	Batch 2: Group	*U*(*N*_Jun-WT_ = 12; *N*_Jun-iCC_ = 12) = 36; *z* = −2.078	*p* *=* 0.038
HB	Social Nosepokes	ANOVA	Group	*F*_(1,29)_ = 0.192	*p* *=* 0.665
HB	Total ambulation	ANOVA	Group	*F*_(1,29)_ = 2.867	*p* *=* 0.101
EPM	Distance	ANOVA	Group	*F*_(1,35)_ = 7.750	*p* *=* 0.009
			Group*sex*batch	*F*_(1,35)_ = 3.370	*p* *=* 0.046
	Open entries	ANOVA	Sex	*F*_(1,35)_ = 6.167	*p* *=* 0.018
			Group*batch	*F*_(1,35)_ = 5.399	*p* *=* 0.009
	Closed entries	ANOVA	Group	*F*_(1,35)_ = 7.125	*p* *=* 0.011
			Group*batch	*F*_(1,35)_ = 6.122	*p* *=* 0.005
	Open time	ANOVA	Sex	*F*_(1,35)_ = 7.564	*p* *=* 0.009
			Group*batch	*F*_(1,35)_ = 3.851	*p* *=* 0.031
	Closed time	ANOVA	Sex	*F*_(1,35)_ = 12.818	*p* *=* 0.001

OF, open field; SMB, sensorimotor battery; HB, odorant holeboard; EPM, elevated plus maze.

### Jun-iCC is relatively silent without neural induction in vivo

**Figure 4.**
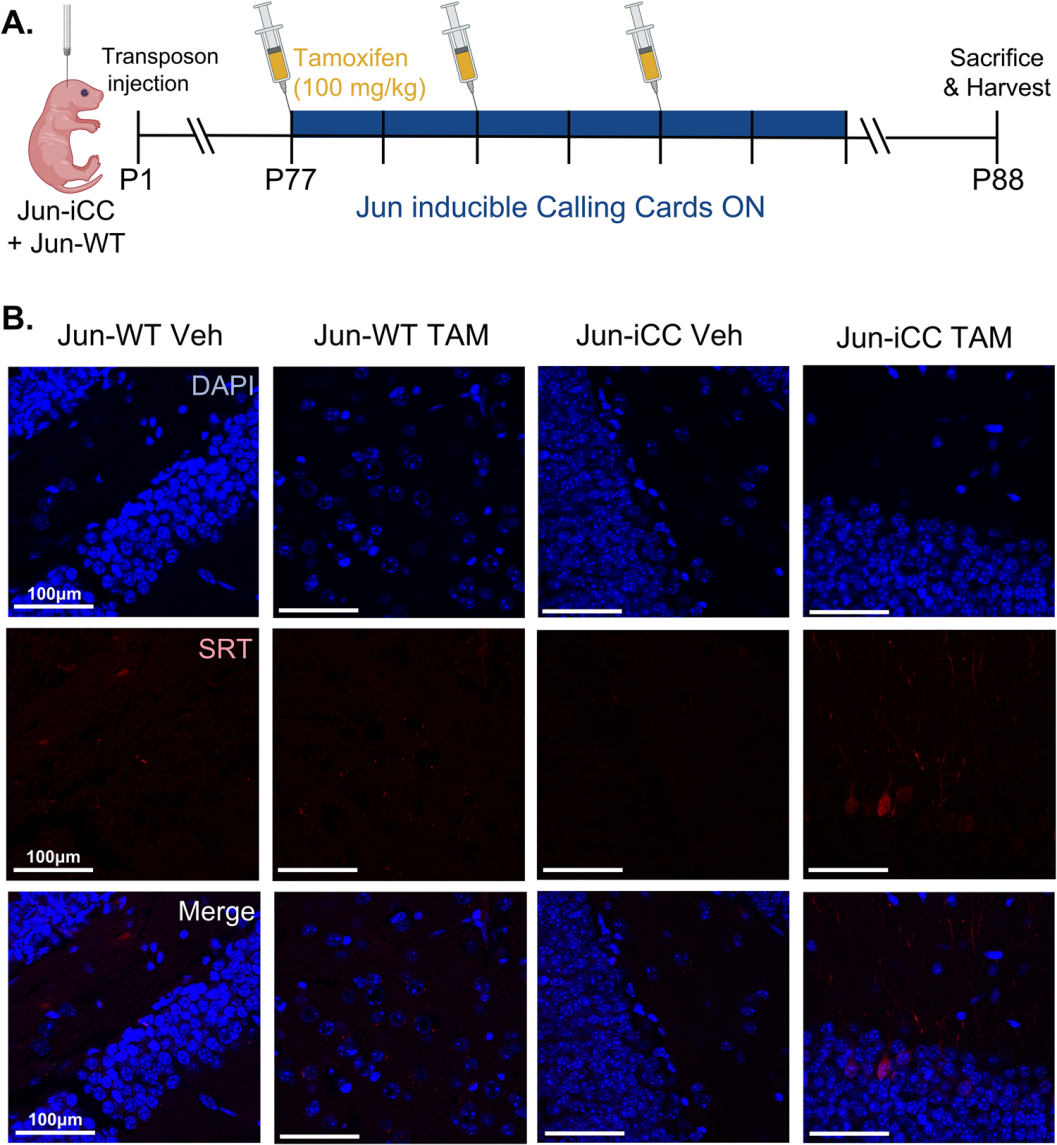
Jun-iCC is silent without neural induction. ***A***, Timeline: Transposon was injected into Jun-iCC (iCC) or Jun-WT (WT) pups at P1. At adulthood (P77), mice were dosed with TAM or vehicle (Veh) for 5 d. The dosing scheme should lead to the TAM presence for 7 d, thus activating Calling Cards recording for 7 d. Mice were killed, and brains were harvested for immunofluorescence 7 d after the last TAM dose. ***B***, Immunofluorescence shows that transposon levels (as measured by RFP fluorescence) were only present at low levels, in the dentate gyrus of the iCC, TAM-dosed animal, suggesting low levels of baseline Jun-iCC activity in the home cage. Scale bar, 100 µM.

Next, we benchmarked the Jun-iCC mouse line in vivo to validate whether it requires induction by both TAM and neural activity. As a “negative control,” we turned on Jun-iCC recording while animals were in the home cage to test if Jun-iCC is neural activity-dependent. At baseline, adult mice have low levels of Jun expression (Ortiz et al., 2020), which should lead to low levels of expression of the Jun-iCC fusion protein and thus low levels of SRT insertions into the DNA as marked by low RFP fluorescence. If Jun-iCC depended on neural activity for recording, we would expect little detectable levels of RFP following a week of recording in the home cage. We tested four groups: Jun-iCC and WT mice, receiving TAM or vehicle (Veh, cornflower oil and ethanol; *n* = 4). We delivered SRTs via AAV9 intracranially to P1 animals, as before (Fig. 4*A*; Cammack et al., 2020). At P77, we dosed mice with TAM at 100 mg/kg every other day, over the course of 5 d (Jahn et al., 2018). This regimen leads to TAM presence in the brain for 7 d, meaning Jun-iCC should be “on” for 7 d. A week after the last TAM dose, at P88, we killed animals and harvested their brains for immunofluorescence, focusing on the dentate gyrus, one of the few regions that is active at baseline (Pofahl et al., 2021). We observed no RFP-positive cells in any WT mice or in Jun-iCC mice that received Veh treatment (Fig. 4*B*). We saw very low levels of RFP-positive cells in the Jun-iCC mice treated with TAM, only in the dentate gyrus, suggesting that Jun-iCC is relatively silent without neural induction in vivo.

### In vivo Jun-iCC recording depends on TAM and neural activity

**Figure 5.**
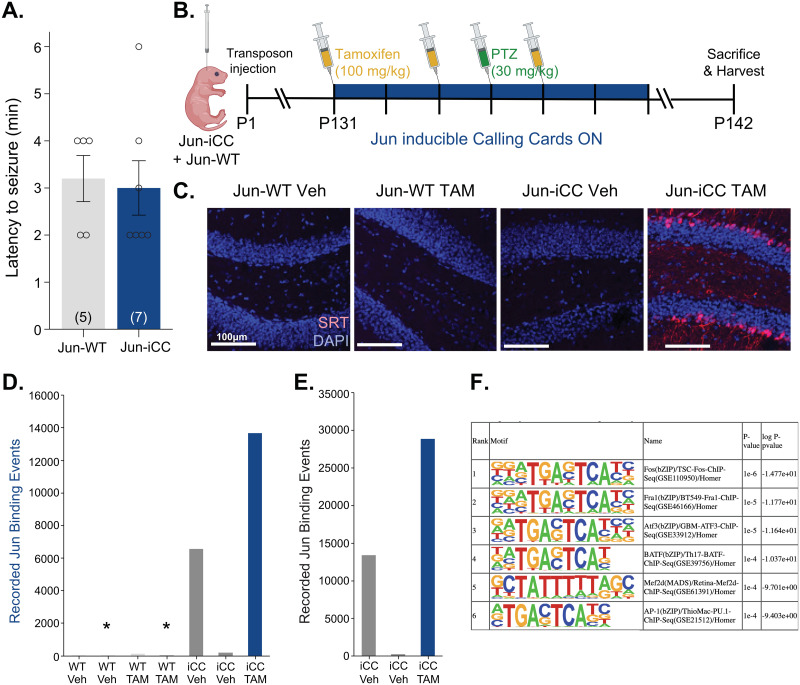
Jun-iCC is induced with TAM and neural activity. ***A***, Jun-iCC (iCC) animals do not show increased susceptibility to seizures relative to Jun- WT littermates. ***B***, Timeline, Transposon was injected into Jun-iCC (iCC) or Jun-WT (WT) pups at P1. At adulthood (P131), mice were dosed with TAM or vehicle (Veh) for 5 d. The dosing scheme should lead to the TAM presence for 7 d, thus activating Calling Cards recording for 7 d. Mice were injected with PTZ to induce low-severity seizures on Day 4 of TAM-induced Calling Cards recording. Mice were killed, and brains were harvested for immunofluorescence and next-generation sequencing 7 d after the last TAM dose. ***C***, Immunofluorescence of the dentate gyrus (DG) shows that only the iCC, TAM-dosed animal had RFP-positive neurons, indicating active Calling Cards recording of PTZ-induced seizures. ***D***, Next-generation sequencing of one-fifth of the brain demonstrated that virtually no Jun binding events were recorded by Calling Cards in the Jun-WT animals regardless of Veh or TAM treatment. Approximately three times more Jun binding events were recovered from the TAM-dosed animal than the average of the two Veh-dosed animals. ***E***, When the entirety of the brain was sequenced for iCC animals only, again approximately three times more Jun binding events were recovered from TAM-dosed than from Veh-dosed animals. ***F***, Homer motif analysis shows significant enrichment for Jun-associated motifs in recovered transposon insertions from sequencing of the entirety of the brain for TAM-dosed iCC animals. Scale bar, 100 µM. Asterisks in bar plots represent samples that did not meet the threshold (>1,000 aligned reads). Additional analyses of Jun-iCC data are presented in Extended Data Figures 5-1 and 5-2.

Given that Jun levels at baseline in the adult mouse brain are low, we need a “positive control” to validate that Jun-iCC is indeed TAM- and activity-dependent. To this end, we induced pharmacological seizures with GABA antagonist PTZ while Jun-iCC recording was on (Van Erum et al., 2019). First, we demonstrate that treating with PTZ (30 mg/kg) induced mild seizures in all animals, and latency to seizure behavior was not impacted by sex, genotype, or TAM treatment (Fig. 5*A*; Racine, 1972; Van Erum et al., 2019). We injected both Jun-iCC and Jun-WT mice (*n* = 7) with SRT on P1. In adulthood, mice were administered TAM or Veh, for 5 d, as above. On Day 4, we delivered 30 mg/kg PTZ intraperitoneally to all animals (Fig. 5*B*) and monitored seizure severity and latency to freezing. All animals exhibiting mild seizures (Racine, 1972; Van Erum et al., 2019), and brains were harvested 8 d after PTZ injection. The left hemisphere of the brain was used for IF for Jun detection, while the right hemisphere was used for RNA sequencing. We found that RFP-positive cells were present only in the dentate gyrus, a region implicated in seizures, in the Jun-iCC TAM animal, with no RFP-positive cells in the other groups (Fig. 5*C*; Erdtmann-Vourliotis et al., 1998; Vasilev et al., 2018). We replicated this experiment in a separate cohort of mice, finding similarly that Jun-iCC TAM-treated mice had a variable but higher area of RFP+ densities in the dentate gyrus, indicating Jun-iCC recording activity, compared with the Jun-iCC Veh-treated and the Jun-WT TAM-treated controls, which had negligible RFP+ density area (Extended Data Fig. 5-2).

10.1523/ENEURO.0411-25.2026.f5-1 Figure 5-1 **Jun inducible Calling Cards is tamoxifen-dependent and targets expected loci *in vitro*. A.** Schematic of how BrokenHeart plasmid/AAV acts as a measure of Calling Card transposase, hyperPiggyBac (hyPB) activity. In brief, BrokenHeart (BH, PRM1294) sequence contains a transposon in between the N- and C-terminals of tdTomato fluorescent reporter. Transcription of native BH leads to non-functional protein, and no red fluorescence. Only in the presence of hyPB activity, with excision of the transposon sequence, can the full tdTomato be transcribed and translated, leading to red fluorescence. **B.** Experimental design of cell culture experiments showing drug exposure for each well condition (TdT: tdTomato transfection, 4-OHT: 4-hydroxytamoxifen 1 μM, propofol 16.8 μM). **C.** Knock-in lines of Jun-inducible Calling Cards show transposon activity, as measured by tdTomato fluorescence from the transposon, almost exclusively in the presence of tamoxifen metabolite (4-OHT). **D.** Quantification of C reveals a ∼10-fold significant induction of RFP by 4-OHT but no further induction by propofol. Note that brightness and contrast were adjusted identically for all images. (n = 8 images per well condition; p < 0.001, One-way ANOVA with post-hoc Tukey HSD test). **E.** Homer motif analysis shows significant enrichment for Jun-associated motifs in transposon insertions from Jun-iCC knock-in lines. Supports Figure 5. Download Figure 5-1, TIF file.

10.1523/ENEURO.0411-25.2026.f5-2 Figure 5-2 **Replication cohort demonstrates Jun-inducible Calling Cards (Jun-iCC) is induced with tamoxifen and neural activity. A.** Timeline: Transposon was injected into Jun-iCC (iCC) or Jun-WT (WT) pups at postnatal day (P) 1. At adulthood (P127/8), mice were dosed with tamoxifen (TAM) or vehicle (Veh) for 5 days. The dosing scheme should lead to tamoxifen presence for 7 days, thus activating Calling Cards recording for 7 days. Mice were injected with pentylenetetrazol (PTZ) to induce low-severity seizures on day 4 of TAM-induced Calling Cards recording. Mice were sacrificed and brains harvested for immunofluorescence 7 days after the last TAM dose, at P140-1. **B.** Immunofluorescence of dentate gyrus (DG) shows that only the two iCC, TAM-dosed animals had RFP-positive neurons, indicating active Calling Cards recording of PTZ-induced seizures. Scale bar: 100 μM. These results from an independent cohort confirm our findings in Figure 5, demonstrating the reproducibility of tamoxifen and activity-dependent recording with the Jun-iCC system across multiple experiments. **C.** RFP + area within the whole image **(B)** measured in pixels shows that some Jun-iCC animals dosed with TAM had larger RFP + areas. Supports Figure 5. Download Figure 5-2, TIF file.

Next, we performed next-generation CC sequencing to identify the number of recorded Jun binding events. The right hemisphere was divided into five samples for technical replicates (Yen et al., 2023). First, we processed only one-fifth of the right hemisphere of all animals. We obtained ∼3× more SRT insertions from the Jun-iCC TAM-dosed animal compared with the two Jun-iCC Veh-dosed animals (Fig. 5*D*; Table 2). Then, we more deeply sampled the Jun-iCC Veh and TAM animals and sequenced their entire right hemisphere. Again, we found ∼3× more recorded Jun binding events in the TAM-dosed relative to the Veh-dosed Jun-iCC mice, with almost 30,000 insertions recorded from the right hemisphere of the Jun-iCC TAM mouse (Fig. 5*E*; Table 3). Jun-WT mice had virtually no insertions regardless of treatment. Notably, 30,000 insertions per hemisphere is an order of magnitude lower than insertions obtained using viral delivery of noninducible hyPB (Cammack et al., 2020). This significant reduction in insertions is likely due to the transient nature of Jun expression in the mouse brain, even after a robust stimulus such as an induced seizure. Finally, we performed Homer motif enrichment analysis (Heinz et al., 2010) on the insertions from the Jun-iCC TAM-treated animal, and we found significant enrichment in Jun and related (AP1, Fos) motifs (Fig. 5*F*), indicating we were marking promoters likely bound by Jun during the seizure.

**Table 2.** Jun-iCC sequencing results table for one-fifth of the brain

Sample ID	Group	Reads	Reads w/ adapter	Reads w/ adapter (%)	Unique alignment	Unique alignment (%)	Insertions	Insertions/Read	Coverage
24324-6	WT Veh	1661	1,628	0.98	932	0.57	6	0.00374	271.33
24324-1	WT TAM	30,381	29,133	0.96	23,968	0.82	137	0.000475	212.65
24324-2	iCC Veh	592,697	549,687	0.93	273,521	0.50	6,568	0.012047	83.69
24324-9	iCC Veh	8,855	8,537	0.96	2,284	0.27	207	0.024	41.24
24324-7	iCC TAM	356,449	328,231	0.92	227,890	0.69	13,668	0.042	24.01

Notably, samples with <1,000 aligned reads were removed.

**Table 3.** Jun-iCC sequencing table for whole brains from positive animals

Sample ID	Group	Reads	Reads w/ adapter	Reads w/ adapter (%)	Unique alignment	Unique alignment (%)	Insertions	Insertions/Read	Coverage
24324-2	iCC Veh	1,102,982	1,038,919	0.942	633,045	0.609	13,426	0.013	77.38
24324-9	iCC Veh	10,794	10,430	0.966	3,497	0.335	256	0.025	40.74
24324-7	iCC TAM	819,042	773,731	0.945	529,806	0.685	28,856	0.037	26.81

### Sp1-iCC is inducible in vitro

Given the relatively low levels of recorded insertions with Jun-iCC, potentially due to its transient expression pattern, we next opted to introduce iCC to a more ubiquitously expressed locus. We chose Sp1 since it is a widely expressed marker of CpG unmethylated regions and thus can serve as a proxy for gene expression (Li et al., 2004; Cammack et al., 2020). Unlike Jun-iCC, which is dependent on both neural activity and TAM for activation, the Sp1-iCC system leverages the constitutive expression of Sp1 across cell types to capture gene expression states more broadly and with higher sensitivity, potentially overcoming the limited recording efficiency we observed with the activity-dependent Jun system.

We engineered an Sp1-iCC knock-in N2a cell line for in vitro validations, with the hyPB-myc-ERT2 knocked into the C-terminus of the endogenous Sp1 locus. Junction PCR and Sanger sequencing of Sp1-hyPB-myc-ERT2 region confirmed that the insertion was successful and there were no mutations in any of the domains. As above, we transfected the BH transposon into Sp1-iCC cells. Using BH, we verified that hyPB insertions happen only in the presence of 4-OHT (Extended Data Fig. 7-1).

### Sp1-iCC negatively impacts viability

**Figure 6.**
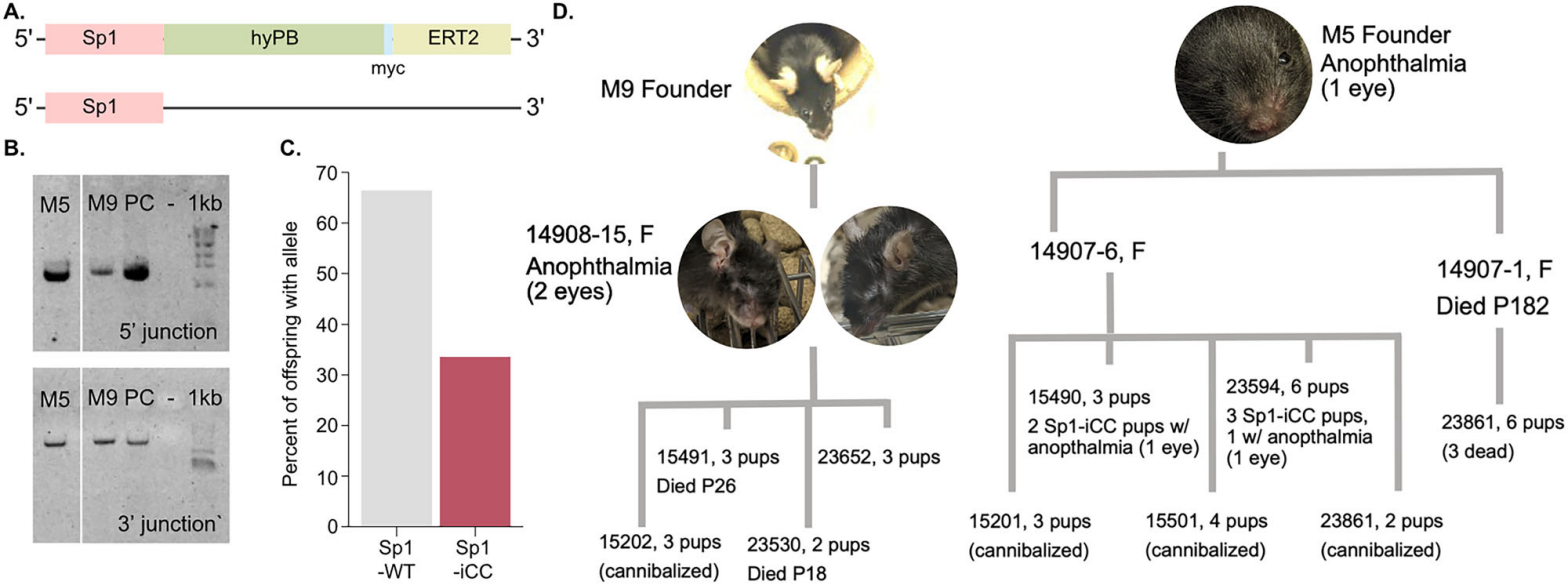
iCC knock into the Sp1 locus is not a viable strategy. ***A***, Schematic of the Sp1 alleles of the Sp1-iCC mouse line. Sp1-iCC had one normal copy of Sp1, and one copy fused to the Calling Card transposase, hyPB, a myc tag, and the TAM-inducible ERT2 domain. ***B***, Junction PCRs showing that both M5 and M9 knock-in lines have expected integration at the Sp1 locus. ***C***, The percentage of offspring (*N* = 131) carrying the Sp1-iCC allele is lower than expected Mendelian 50%, potentially indicating reduced viability of Sp1-iCC pups relative to Sp1-WT pups. ***D***, Representative family tree of litters from two separate founder lines indicating widespread presence of anophthalmia, small litters, early loss of pups, and mortality around weaning age, overall suggesting limited viability of the Sp1-iCC mouse lines. Enlarged images shown in this figure are presented in Extended Data Figure 6-1.

We then knocked in the Sp1-iCC construct into mouse embryos to generate Sp1-iCC founder mice, with hyPB-myc-ERT2 knocked into the endogenous Sp1 locus (Fig. 6*A*). We performed junction PCRs to confirm insertion sites and Sanger sequencing to verify no mutations were introduced (Fig. 6*B*). We generated two founder lines, M5 and M9. Offspring of both lines did not show expected Mendelian ratios, with only ∼30% of total live offspring being Sp1-iCC heterozygous, compared with the expected 50%, suggesting that the Sp1-iCC knock-in negatively impacts viability (Fig. 6*C*). Indeed, when looking at offspring from both founder lines, we saw noticeable reductions in litter size from the expected 6–8 pups per litter and increased death of pups immediately after birth or around weaning (Fig. 6*D*; Finlay et al., 2015). We also noticed aberrant phenotypes in the founders and offspring, with anophthalmia being the most prominent

10.1523/ENEURO.0411-25.2026.f6-1 Figure 6-1 **Full-sized versions of the SP1 mouse photos from panel 6D. A.** M9 Founder. **B.** 14908-15 (F) with anophthalmia. **(C & D)** M5 Founder with anophthalmia. Supports Figure 6. Download Figure 6-1, TIF file.

### In vivo Sp1-iCC recording depends on TAM

**Figure 7.**
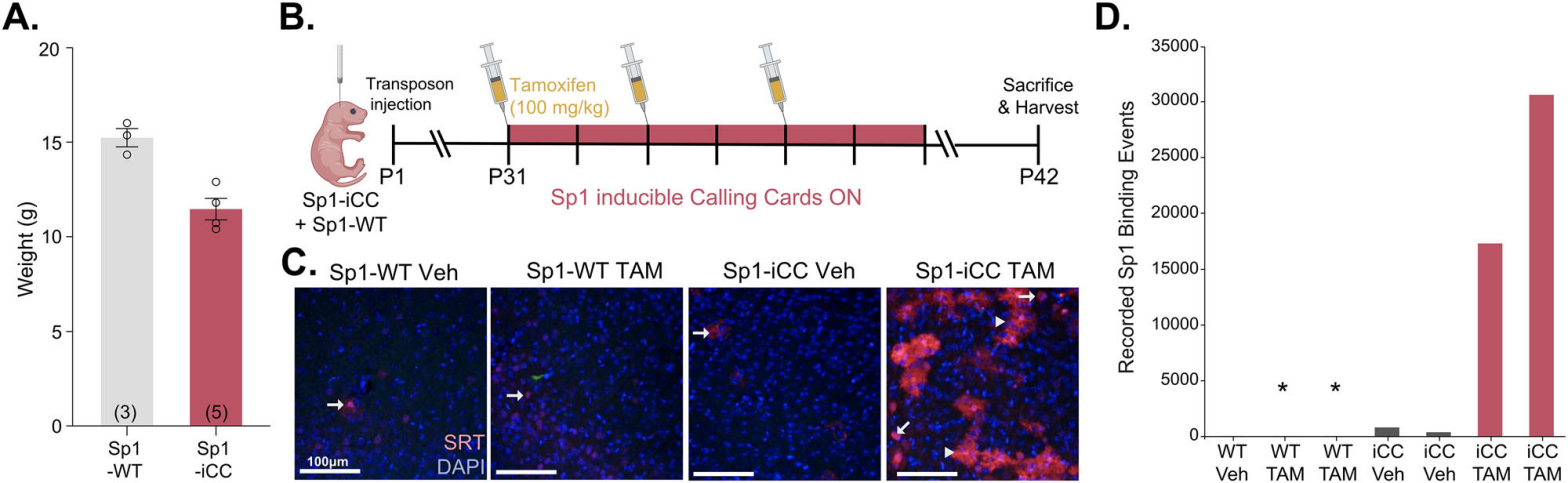
Sp1-inducible Calling Cards (Jun-iCC) is induced with TAM. ***A***, Sp1-iCC (iCC) animals weigh less than Sp1-WT littermates (*p* = 0.005). ***B***, Timeline, Transposon was injected into Sp1-iCC (iCC) or Sp1-WT (WT) pups at P1. At juvenile age (P31), mice were dosed with TAM or vehicle (Veh) for 5 d. The dosing scheme should lead to the TAM presence for 7 d, thus activating Calling Cards recording for 7 d. Mice were killed, and brains were harvested for immunofluorescence and next-generation sequencing 7 d after the last TAM dose. ***C***, Immunofluorescence of the cortex shows that only the iCC, TAM-dosed animal had RFP-positive cells, including some with morphology of neurons (arrows) and astrocytes (arrowheads) indicating active Calling Cards recording of Sp1 binding. ***D***, Next-generation sequencing of one-fifth of the brain demonstrated that virtually no Sp1 binding events were recorded by Calling Cards in the WT animals, with tens of thousands of insertions recovered from the TAM-dosed animals. Scale bar, 100 M. Asterisks in bar plots represent samples that did not meet the threshold (>1,000 aligned reads). Additional analyses of Sp1-iCC data are presented in Extended Data Figures 7-1 and 7-2.

To test whether Sp1-iCC recording in vivo was TAM-inducible, we tested four groups: Sp1-iCC and WT mice (*n* = 7), receiving TAM or Veh, for 5 d, as with Jun-iCC above (Fig. 7*B*). All mice had received SRT injections intracranially on P1. We killed animals a week after their last dose and harvested brains, with the left hemisphere for IF and the right hemisphere for sequencing. We found that RFP-positive cells, including in cells with the morphology of both neurons and astrocytes, were only present in the cortex of Sp1-iCC TAM mice, with no RFP-positive cells in the other groups (Fig. 7*C*). We replicated this experiment in a separate cohort of mice, finding similarly that only the Sp1-iCC TAM-treated mice had a noticeably higher number of RFP+ neurons in the cortex as well as the dentate gyrus, with negligible levels of RFP+ neurons in the brains of control littermates (Extended Data Fig. 7-2).

10.1523/ENEURO.0411-25.2026.f7-1 Figure 7-1 **Sp1 inducible Calling Cards is tamoxifen-dependent *in vitro*. A.** Experimental design to test the Sp1 line (tdT: tdTomato transfection, NC: negative control, 4-OHT: 1 μM). **B**. Representative images of bright field (BF) and RFP with high or low contrast. Note that brightness and contrast were adjusted identically for all images. **C.** Quantification showing impact of 4-OHT treatment, n = 8 images per well condition; p < 0.01, One-way ANOVA with post-hoc Tukey HSD test. Scale bar: 100 μM. Supports Figure 7. Download Figure 7-1, TIF file.

10.1523/ENEURO.0411-25.2026.f7-2 Figure 7-2 **Replication cohort demonstrates Sp1-inducible Calling Cards (Sp1-iCC) is induced with tamoxifen. A.** Timeline: Transposon was injected into Sp1-iCC (iCC) or Sp1-WT (WT) pups at postnatal day (P)1. At juvenile age (P31), mice were dosed with tamoxifen (TAM) or vehicle (Veh) for 5 days. The dosing scheme should lead to tamoxifen presence for 7 days, thus activating Calling Cards recording for 7 days. Mice were sacrificed and brains harvested for immunofluorescence 7 days after the last TAM dose. **B.** Immunofluorescence of cortex (top panels) and dentate gyrus (bottom panels) shows that only the iCC, TAM-dosed animals had RFP-positive neurons, indicating active Calling Cards recording of PTZ-induced seizures. **(C & D)** RFP + area within the whole image **(B)** measured in pixels shows that some Jun-iCC animals dosed with TAM had larger RFP + areas within the cortex and dentate gyrus, respectively. Scale bar: 100 μM. Supports Figure 7. Download Figure 7-2, TIF file.

Finally, we performed next-generation sequencing in the first cohort to identify the number of recorded Sp1 binding events in one-fifth of the right hemisphere of all animals. We obtained no SRT insertions in the Sp1-WT animals and negligible amounts of SRT insertions in the Veh-dosed Sp1-iCC animals. The Sp1-iCC TAM-dosed animals had robust recording of Sp1 binding events in these animals (Fig. 7*D*; Table 4). Notably we obtained over 30,000 insertions from only a fifth of a hemisphere from Sp1-iCC, compared with ∼30,000 from a full hemisphere with Jun-iCC, suggesting that Sp1-iCC does express more ubiquitously and thus leads to much more recording. Still, while Sp1-iCC provides superior recording efficiency, the developmental impacts in this mouse line will make it challenging to use for many applications.

**Table 4.** Sp1-iCC sequencing table for whole brains

Sample ID	Group	Reads	Reads w/ adapter	Reads w/ adapter (%)	Unique alignment	Unique alignment (%)	Insertions	Insertions/Read	Coverage
15490-4	WT Veh	18,619	1,662	8.93	55	3.31	24	0.014	69.25
15490-7	iCC Veh	8,050	1,867	23.19	1,578	84.52	814	0.44	2.29
15490-3	iCC Veh	11,505	1,043	9.066	931	89.26	373	0.36	2.80
15490-2	iCC TAM	101,731	79,111	77.76	72,289	91.38	17,320	0.22	4.57
15490-6	iCC TAM	108,159	89,729	82.96	79,803	88.94	30,709	0.34	2.92

Notably, samples with <1,000 aligned reads were removed.

## Discussion

Our study presents the development and characterization of iCC technology, a novel approach for temporally controlled recording of TF-DNA binding events in vivo. We engineered two distinct mouse lines targeting different TFs: Jun-iCC and Sp1-iCC, each revealing important biological constraints and potential applications for molecular recording in neuroscience.

The Jun-iCC system successfully demonstrated TAM-dependent inducibility both in vitro and in vivo, with low-background recording in the absence of TAM or neural activity. These mice showed normal development and behavior across multiple domains, including locomotion, sensorimotor function, anxiety-related behaviors, and olfaction. When activated by TAM during PTZ-induced seizures, Jun-iCC effectively tagged activated neurons with RFP and recorded ∼30,000 insertions per hemisphere. The recorded insertions are lower than expected for nonactivity-dependent CC tools (Cammack et al., 2020; Yen et al., 2023; Boros et al., 2025), likely due to the transient expression pattern of IEGs in the brain. While this recording efficiency may be sufficient for TRAP-like approaches to mapping circuit activation, it appears insufficient for comprehensive epigenetic profiling of Jun binding unless 10-fold more mice were used (as hundreds of thousands of insertions would be required to facilitate peak calling).

In contrast, the Sp1-iCC system provided substantially higher recording efficiency, yielding over 30,000 insertions from just one-fifth of a hemisphere. This confirms our hypothesis that targeting a ubiquitously expressed TF would enhance recording capabilities. However, this came at a significant cost to viability. Sp1-iCC mice exhibited reduced Mendelian ratios (∼30% instead of the expected 50%), developmental abnormalities including anophthalmia, reduced body weight, and high mortality rates among offspring.

Interestingly, the severe developmental impact of Sp1-iCC suggests that Sp1 heterozygosity may be functionally lethal or severely detrimental, which aligns with previous literature on Sp1 heterozygosity, where heterozygous mice also display anophthalmia in one or both eyes and growth restriction, as well as decreased erythroid progenitor cells (Marin et al., 1997; Krüger et al., 2007). However, our added subviability suggests that Sp1-iCC may even have some dominant negative activity that makes it more severe than heterozygous loss of function, perhaps sequestering key Sp1 binding partners outside the nucleus. Our findings provide further insight into Sp1's essential role in development. In contrast, Jun-iCC mice exhibited normal development and behavior, consistent with observations from other IEG-inducible models such as Fos-TRAP and Arc-TRAP (Guenthner et al., 2013). This strengthens the suggestion that a single functional copy of IEGs is sufficient for normal development, potentially due to redundancy in their gene function.

The Jun-iCC system demonstrates that IEGs derived from transposons can effectively mark activated neurons with fluorescent proteins, akin to Fos-TRAP. Detection of fluorescence requires relatively few copies of tdTomato mRNA to visualize a cell, making it suitable for cellular labeling. However, comprehensive transcriptional recording requires hundreds of thousands of insertions per mouse to be applicable for downstream analysis, which is orders of magnitude more than what is currently achievable with transiently expressed factors like Jun (Yen et al., 2023). Nevertheless, this approach might still prove valuable in tissues where Jun expression is more sustained, such as DRGs, where recording efficiency could reach levels suitable for molecular analysis (Buschmann et al., 1998). Likewise, the current iteration may be sufficient to provide an orthogonal or complementary approach to TRAP but with reporters and other transgenes delivered by transposon rather than LoxP-mediated recombination.

One limitation of the CreERT2 system used here is that it sequesters Cre in the cytosol via binding to heat shock proteins until the primary metabolite of TAM induces a conformational change, allowing the Cre to translocate to the nucleus. However, like all CreERT2 systems, this system is subject to basal translocation given that heat shock protein binding is not 100% efficient, and levels of naturally occurring estradiols and cell-type specific nuclear transport mechanisms can vary between cell types, facilitating some possibility for spontaneous translocation.

For future iterations of iCC technology, the most promising approach would be to target a nonendogenous locus, such as the Rosa26 locus, with either a ubiquitous TF fused to hyPB or the native hyPB, which has natural affinity for BRD4 at super enhancer sites (Chu et al., 2016; Cammack et al., 2020; Moudgil et al., 2020). Such a construct would avoid the developmental consequences of disrupting essential endogenous genes while maintaining temporal control through the ERT2 domain. In this scenario, using just the Sp1 promoter region or first exon to drive expression and knocking this construct into the Rosa26 locus, rather than modifying the endogenous Sp1 locus itself, could provide the efficiency benefits of Sp1-driven expression without the detrimental impacts on viability.

A currently untestable question in behavioral neuroscience is why genetically identical, similarly reared littermate mice dichotomize into susceptible versus resilient groups in various models, including chronic social defeat stress (CSDS), addiction, and social motivation (Krishnan et al., 2007; Maloney et al., 2023; Slivicki et al., 2023). In the well-studied CSDS model, resilient and susceptible groups demonstrate significantly different transcriptional profiles at sacrifice (Krishnan et al., 2007). Hence, the leading hypothesis is that resilience is predicated by differing molecular landscape from susceptibility mice prior to the stressor (Nestler and Waxman, 2020). To test this hypothesis, a tool to precisely record transcriptional state in a limited window prior to behavioral manipulation and then stop recording during the behavioral manipulation would be ideal. Thus, further updates of the iCC system in vivo would be of interest.

Overall, our development of iCC technology successfully demonstrates the potential for temporally controlled recording of transcriptional states in vivo while highlighting important biological constraints when implementing such systems with specific TFs, such as Jun and Sp1. These mice are no longer live for further current experimentation, but frozen sperm has been nominated to Jackson labs/MNMRC for distribution to other labs interested in using this current generation of iCC tools. Future iterations should consider utilizing the endogenous hyPB with its natural affinity for BRD4 to capture super enhancer sites or alternative ubiquitous TFs delivered exogenously rather than knocked into endogenous loci, which may avoid the developmental consequences of sequestering essential genes.

## Synthesis

Reviewing Editor: Benjamin Deneen, Baylor College of Medicine Decisions are customarily a result of the Reviewing Editor and the peer reviewers coming together and discussing their recommendations until a consensus is reached. When revisions are invited, a fact-based synthesis statement explaining their decision and outlining what is needed to prepare a revision will be listed below. The following reviewer(s) agreed to reveal their identity: NONE.

We had a difficult time attaining reviewers for this manuscript

I have reviewed the paper and generally agree with the thorough comments and revisions recommended by the reviewer.
