# Supplementary figures and images for: Inducible Calling Cards: Developing Mouse Reagents for Experimentally Controlled Transposon Insertion In Vivo
Source: eNeuro. 2026 Jun 24;13(6):ENEURO.0411-25.2026. doi: 10.1523/ENEURO.0411-25.2026 (PMC13421841; doi:10.1523/ENEURO.0411-25.2026)

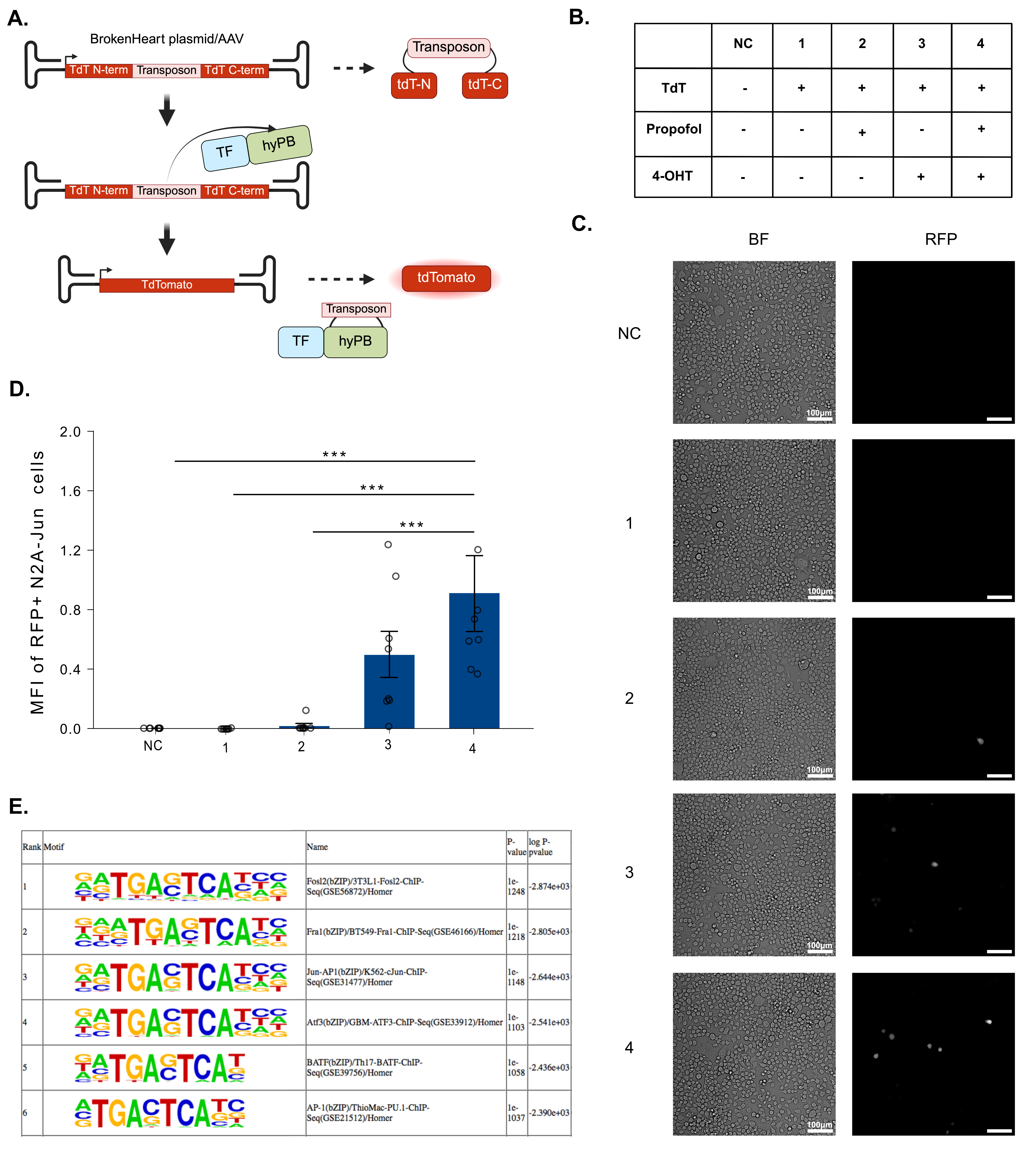

Supplement: Figure 5-1 — Jun inducible Calling Cards is tamoxifen-dependent and targets expected loci in vitro. A. Schematic of how BrokenHeart plasmid/AAV acts as a measure of Calling Card transposase, hyperPiggyBac (hyPB) activity. In brief, BrokenHeart (BH, PRM1294) sequence contains a transposon in between the N- and C-terminals of tdTomato fluorescent reporter. Transcription of native BH leads to non-functional protein, and no red fluorescence. Only in the presence of hyPB activity, with excision of the transposon sequence, can the full tdTomato be transcribed and translated, leading to red fluorescence. B. Experimental design of cell culture experiments showing drug exposure for each well condition (TdT: tdTomato transfection, 4-OHT: 4-hydroxytamoxifen 1 μM, propofol 16.8 μM). C. Knock-in lines of Jun-inducible Calling Cards show transposon activity, as measured by tdTomato fluorescence from the transposon, almost exclusively in the presence of tamoxifen metabolite (4-OHT). D. Quantification of C reveals a ∼10-fold significant induction of RFP by 4-OHT but no further induction by propofol. Note that brightness and contrast were adjusted identically for all images. (n = 8 images per well condition; p < 0.001, One-way ANOVA with post-hoc Tukey HSD test). E. Homer motif analysis shows significant enrichment for Jun-associated motifs in transposon insertions from Jun-iCC knock-in lines. Supports Figure 5. Download Figure 5-1, TIF file. [file eneuro-13-ENEURO.0411-25.2026-s002.tif]

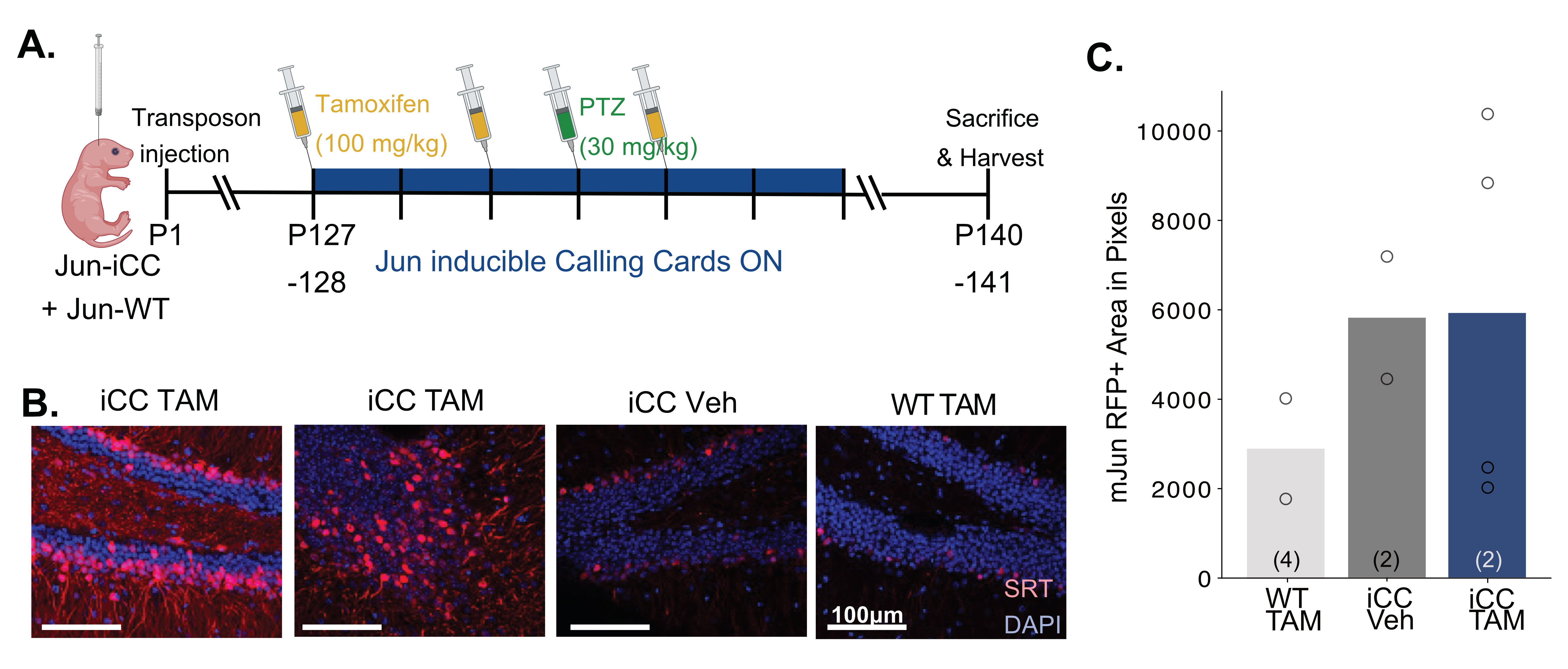

Supplement: Figure 5-2 — Replication cohort demonstrates Jun-inducible Calling Cards (Jun-iCC) is induced with tamoxifen and neural activity. A. Timeline: Transposon was injected into Jun-iCC (iCC) or Jun-WT (WT) pups at postnatal day (P) 1. At adulthood (P127/8), mice were dosed with tamoxifen (TAM) or vehicle (Veh) for 5 days. The dosing scheme should lead to tamoxifen presence for 7 days, thus activating Calling Cards recording for 7 days. Mice were injected with pentylenetetrazol (PTZ) to induce low-severity seizures on day 4 of TAM-induced Calling Cards recording. Mice were sacrificed and brains harvested for immunofluorescence 7 days after the last TAM dose, at P140-1. B. Immunofluorescence of dentate gyrus (DG) shows that only the two iCC, TAM-dosed animals had RFP-positive neurons, indicating active Calling Cards recording of PTZ-induced seizures. Scale bar: 100 μM. These results from an independent cohort confirm our findings in Figure 5, demonstrating the reproducibility of tamoxifen and activity-dependent recording with the Jun-iCC system across multiple experiments. C. RFP + area within the whole image (B) measured in pixels shows that some Jun-iCC animals dosed with TAM had larger RFP + areas. Supports Figure 5. Download Figure 5-2, TIF file. [file eneuro-13-ENEURO.0411-25.2026-s003.tif]

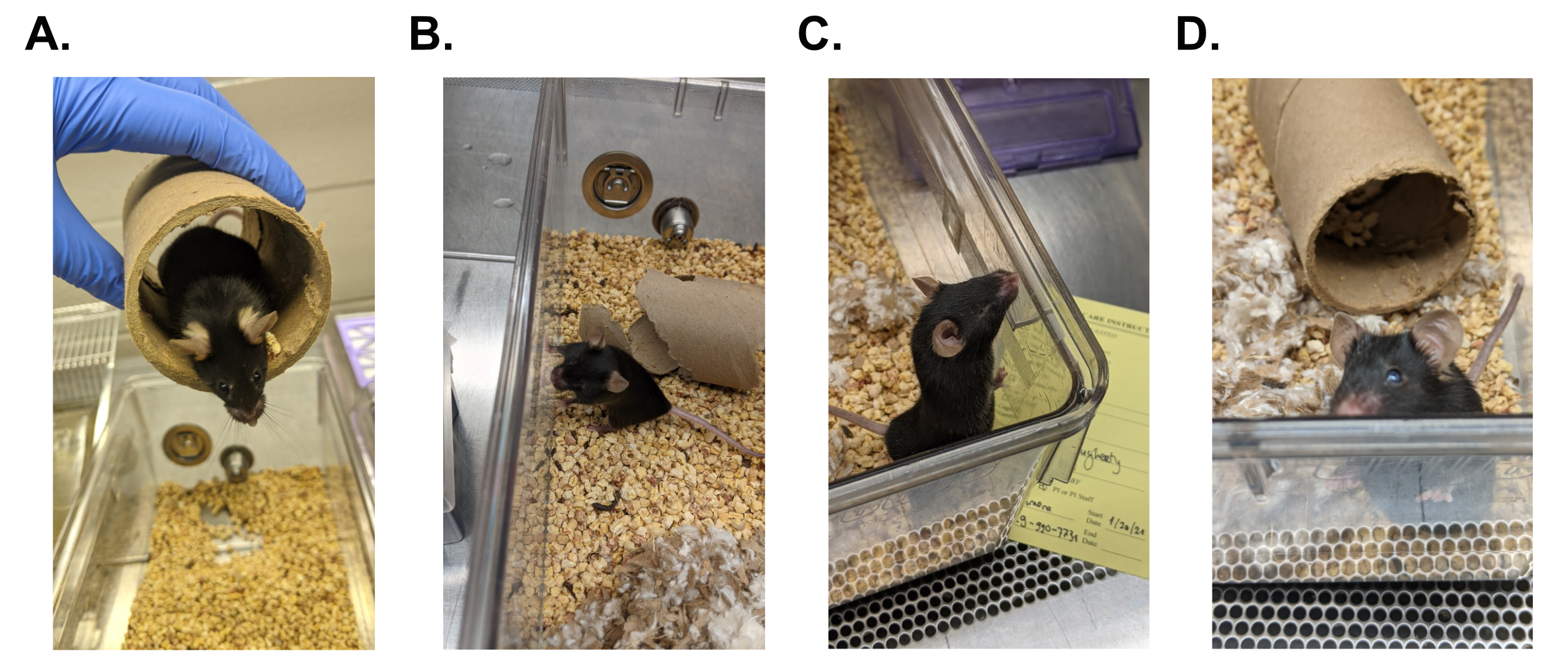

Supplement: Figure 6-1 — Full-sized versions of the SP1 mouse photos from panel 6D. A. M9 Founder. B. 14908-15 (F) with anophthalmia. (C & D) M5 Founder with anophthalmia. Supports Figure 6. Download Figure 6-1, TIF file. [file eneuro-13-ENEURO.0411-25.2026-s004.tif]

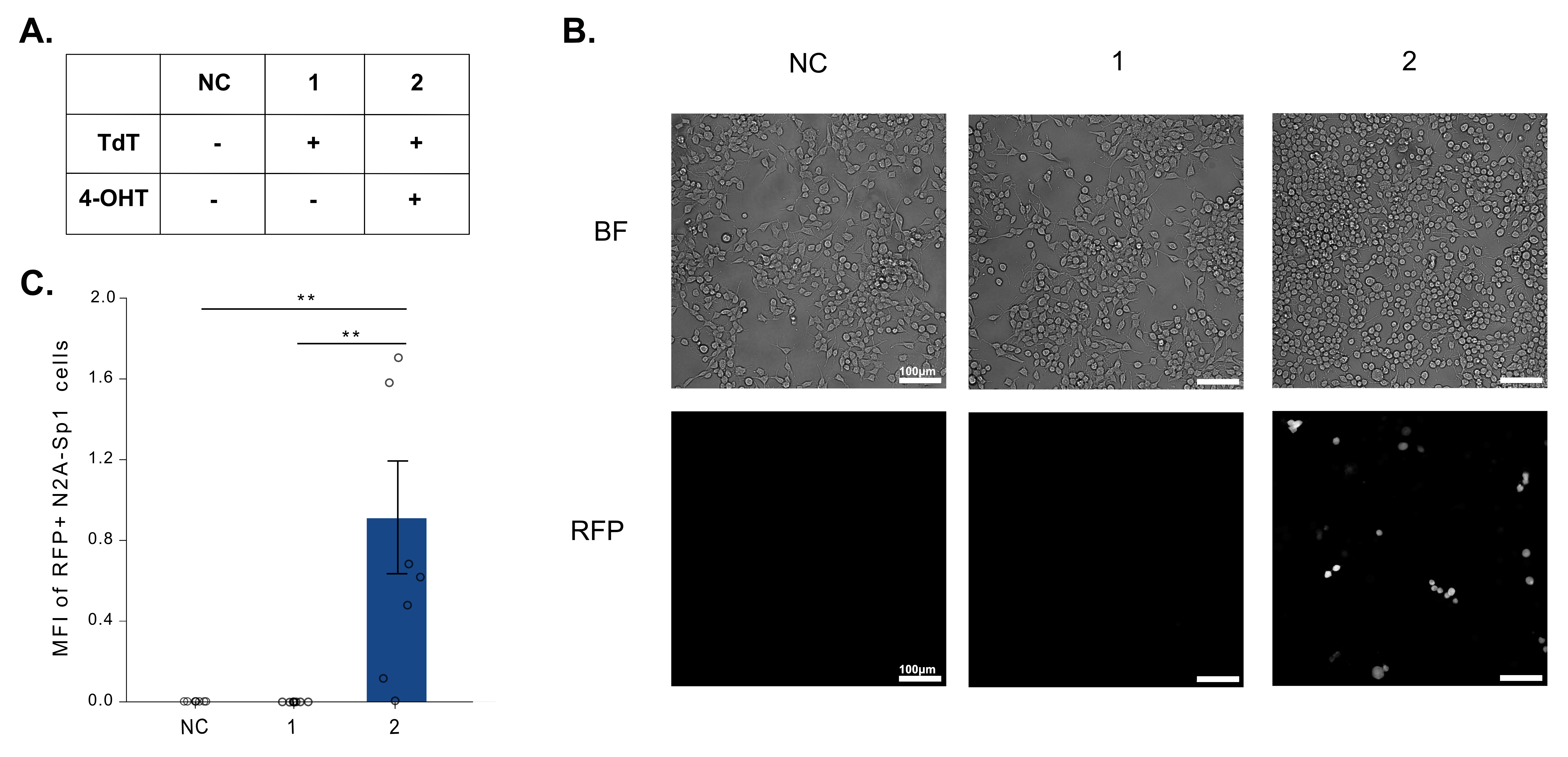

Supplement: Figure 7-1 — Sp1 inducible Calling Cards is tamoxifen-dependent in vitro. A. Experimental design to test the Sp1 line (tdT: tdTomato transfection, NC: negative control, 4-OHT: 1 μM). B. Representative images of bright field (BF) and RFP with high or low contrast. Note that brightness and contrast were adjusted identically for all images. C. Quantification showing impact of 4-OHT treatment, n = 8 images per well condition; p < 0.01, One-way ANOVA with post-hoc Tukey HSD test. Scale bar: 100 μM. Supports Figure 7. Download Figure 7-1, TIF file. [file eneuro-13-ENEURO.0411-25.2026-s005.tif]

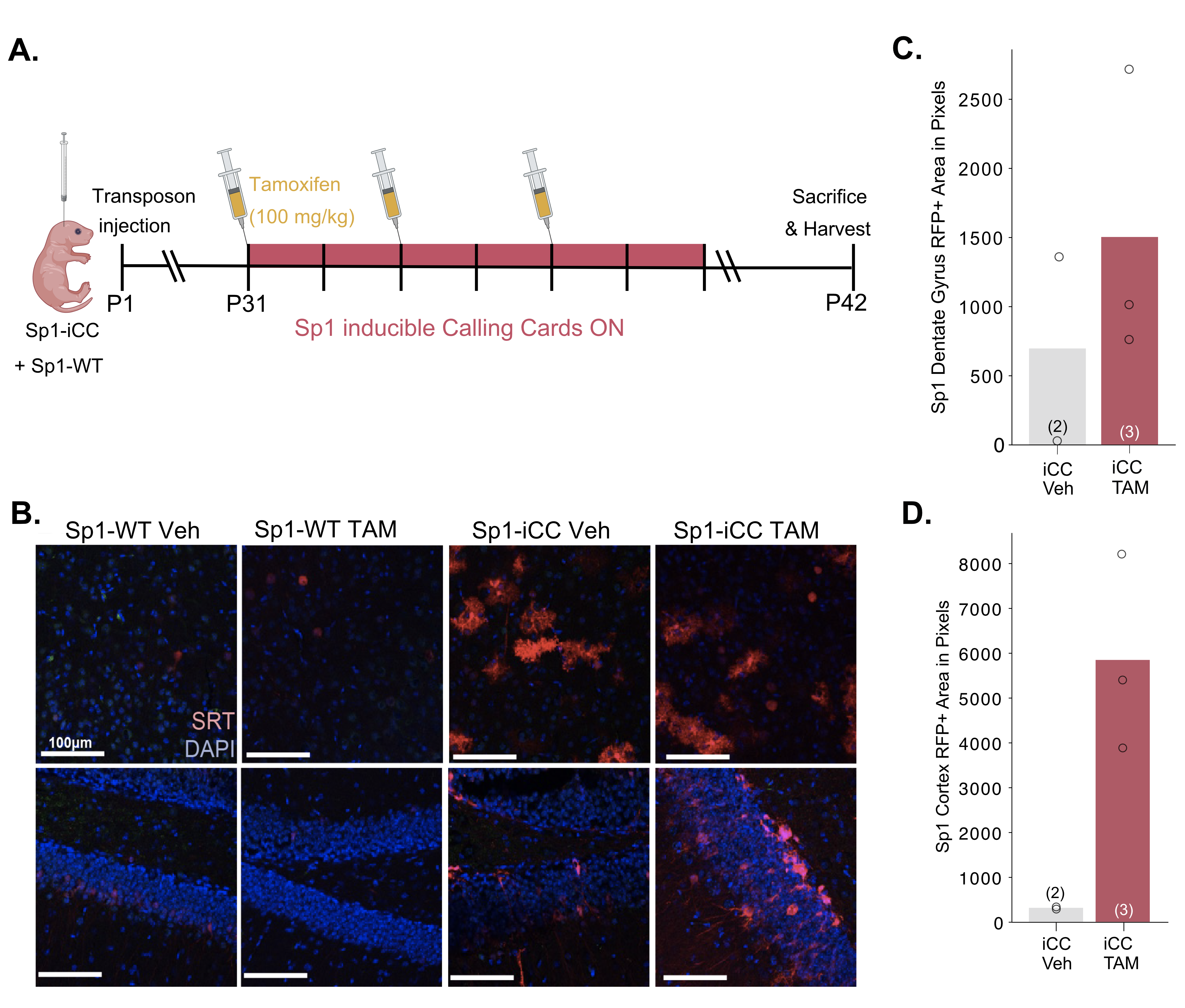

Supplement: Figure 7-2 — Replication cohort demonstrates Sp1-inducible Calling Cards (Sp1-iCC) is induced with tamoxifen. A. Timeline: Transposon was injected into Sp1-iCC (iCC) or Sp1-WT (WT) pups at postnatal day (P)1. At juvenile age (P31), mice were dosed with tamoxifen (TAM) or vehicle (Veh) for 5 days. The dosing scheme should lead to tamoxifen presence for 7 days, thus activating Calling Cards recording for 7 days. Mice were sacrificed and brains harvested for immunofluorescence 7 days after the last TAM dose. B. Immunofluorescence of cortex (top panels) and dentate gyrus (bottom panels) shows that only the iCC, TAM-dosed animals had RFP-positive neurons, indicating active Calling Cards recording of PTZ-induced seizures. (C & D) RFP + area within the whole image (B) measured in pixels shows that some Jun-iCC animals dosed with TAM had larger RFP + areas within the cortex and dentate gyrus, respectively. Scale bar: 100 μM. Supports Figure 7. Download Figure 7-2, TIF file. [file eneuro-13-ENEURO.0411-25.2026-s006.tif]
